# Immunogenicity of standard and extended dosing intervals of BNT162b2 mRNA vaccine

**DOI:** 10.1016/j.cell.2021.10.011

**Published:** 2021-11-11

**Authors:** Rebecca P. Payne, Stephanie Longet, James A. Austin, Donal T. Skelly, Wanwisa Dejnirattisai, Sandra Adele, Naomi Meardon, Sian Faustini, Saly Al-Taei, Shona C. Moore, Tom Tipton, Luisa M. Hering, Adrienn Angyal, Rebecca Brown, Alexander R. Nicols, Natalie Gillson, Susan L. Dobson, Ali Amini, Piyada Supasa, Andrew Cross, Alice Bridges-Webb, Laura Silva Reyes, Aline Linder, Gurjinder Sandhar, Jonathan A. Kilby, Jessica K. Tyerman, Thomas Altmann, Hailey Hornsby, Rachel Whitham, Eloise Phillips, Tom Malone, Alexander Hargreaves, Adrian Shields, Ayoub Saei, Sarah Foulkes, Lizzie Stafford, Sile Johnson, Daniel G. Wootton, Christopher P. Conlon, Katie Jeffery, Philippa C. Matthews, John Frater, Alexandra S. Deeks, Andrew J. Pollard, Anthony Brown, Sarah L. Rowland-Jones, Juthathip Mongkolsapaya, Eleanor Barnes, Susan Hopkins, Victoria Hall, Christina Dold, Christopher J.A. Duncan, Alex Richter, Miles Carroll, Gavin Screaton, Thushan I. de Silva, Lance Turtle, Paul Klenerman, Susanna Dunachie, on behalf of the PITCH Consortium, Hibatullah Abuelgasim, Emily Adland, Syed Adlou, Hossain Delowar Akther, Ahmed Alhussni, Mohammad Ali, M. Azim Ansari, Carolina V. Arancibia-Cárcamo, Martin Bayley, Helen Brown, Jeremy Chalk, Meera Chand, Anu Chawla, Senthil Chinnakannan, Jospeh Cutteridge, Catherine de Lara, Lucy Denly, Ben Diffey, Stavros Dimitriadis, Thomas M. Drake, Timothy Donnison, Maeva Dupont, David Eyre, Alex Fairman, Siobhan Gardiner, Javier Gilbert-Jarmillo, Philip Goulder, Carl-Philipp Hackstein, Sophie Hambleton, Muzlifah Haniffa, Jenny Haworth, Jennifer Holmes, Emily Horner, Anni Jämsén, Sile Johnson, Chris Jones, Mwila Kasanyinga, Sinead Kelly, Rosemary Kirk, Michael L. Knight, Allan Lawrie, Lian Lee, Lauren Lett, Katy Lillie, Nicholas Lim, Hema Mehta, Alexander J. Mentzer, Denise O’Donnell, Ane Ogbe, Matthew Pace, Brendan A.I. Payne, Gareth Platt, Sonia Poolan, Nicholas Provine, Narayan Ramamurthy, Nichola Robinson, Leigh Romaniuk, Patpong Rongkard, Oliver L. Sampson, Beatrice Simmons, Jarmila S. Spegarova, Emily Stephenson, Kris Subramaniam, James Thaventhiran, Sarah Thomas, Simon Travis, Stephanie Tucker, Helena Turton, Adam Watson, Lisa Watson, Esme Weeks, Robert Wilson, Steven Wood, Rachel Wright, Huiyuan Xiao, Amira A.T. Zawia

**Affiliations:** 1 Translational and Clinical Research Institute Immunity and Inflammation Theme, Newcastle University, Newcastle, UK; 2 Wellcome Centre for Human Genetics, Nuffield Department of Medicine, University of Oxford, Oxford, UK; 3 NIHR Health Protection Research Unit in Emerging and Zoonotic Infections, Institute of Infection, Veterinary and Ecological Sciences, University of Liverpool, Liverpool, UK; 4 Peter Medawar Building for Pathogen Research, Nuffield Department of Clinical Medicine, University of Oxford, Oxford, UK; 5 Oxford University Hospitals NHS Foundation Trust, Oxford, UK; 6 Nuffield Department of Clinical Neuroscience, University of Oxford, Oxford, UK; 7 Sheffield Teaching Hospitals NHS Foundation Trust, Sheffield, UK; 8 Institute of Immunology and Immunotherapy, College of Medical and Dental Science, University of Birmingham, Birmingham, UK; 9 Department of Infection, Immunity and Cardiovascular Disease, University of Sheffield, Sheffield, UK; 10 Public Health England, Colindale, London, UK; 11 Translational Gastroenterology Unit, University of Oxford, Oxford, UK; 12 Liverpool University Hospitals NHS Foundation Trust, Liverpool, UK; 13 Oxford Vaccine Group, Department of Paediatrics, University of Oxford, Oxford, UK; 14 Great North Children’s Hospital, Newcastle, UK; 15 University Hospitals Birmingham NHS Foundation Trust, Birmingham, UK; 16 Oxford University Medical School, Medical Sciences Division, University of Oxford, Oxford, UK; 17 Institute of Infection, Veterinary and Ecological Sciences, University of Liverpool, Liverpool, UK; 18 Oxford Centre For Global Health Research, Nuffield Department of Clinical Medicine, University of Oxford, Oxford, UK; 19 Radcliffe Department of Medicine, University of Oxford, Oxford, UK; 20 NIHR Oxford Biomedical Research Centre, University of Oxford, Oxford, UK; 21 Chinese Academy of Medical Science (CAMS) Oxford Institute (COI), University of Oxford, Oxford, UK; 22 Siriraj Center of Research Excellence in Dengue & Emerging Pathogens, Faculty of Medicine Siriraj Hospital, Mahidol University, Bangkok, Thailand; 23 Faculty of Medicine, Department of Infectious Disease, Imperial College London, London, UK; 24 NIHR Health Protection Research Unit in Healthcare Associated Infection and Antimicrobial Resistance, University of Oxford, Oxford, UK; 25 Department of Infection and Tropical Medicine, Newcastle upon Tyne Hospitals NHS Foundation Trust, Newcastle, UK; 26 Mahidol-Oxford Tropical Medicine Research Unit, Bangkok, Thailand

**Keywords:** SARS-CoV-2, COVID-19, vaccine, BNT162b2, antibody, neutralization, B cell, T cell, dosing interval, variants of concern

## Abstract

Extension of the interval between vaccine doses for the BNT162b2 mRNA vaccine was introduced in the United Kingdom to accelerate population coverage with a single dose. At this time, trial data were lacking, and we addressed this in a study of United Kingdom healthcare workers. The first vaccine dose induced protection from infection from the circulating alpha (B.1.1.7) variant over several weeks. In a substudy of 589 individuals, we show that this single dose induces severe acute respiratory syndrome coronavirus 2 (SARS-CoV-2) neutralizing antibody (NAb) responses and a sustained B and T cell response to the spike protein. NAb levels were higher after the extended dosing interval (6–14 weeks) compared with the conventional 3- to 4-week regimen, accompanied by enrichment of CD4^+^ T cells expressing interleukin-2 (IL-2). Prior SARS-CoV-2 infection amplified and accelerated the response. These data on dynamic cellular and humoral responses indicate that extension of the dosing interval is an effective immunogenic protocol.

## Highlights

• BNT162b2 vaccine with an extended interval between doses is highly protective
• Antibody levels were higher after the extended regimen compared with the short regimen
• The extended regimen enriches for virus-specific CD4^+^ T cells expressing IL-2
• Antibody levels wane after each dose, but B and T cell pools are maintained

## Graphical abstract

**Figure:**
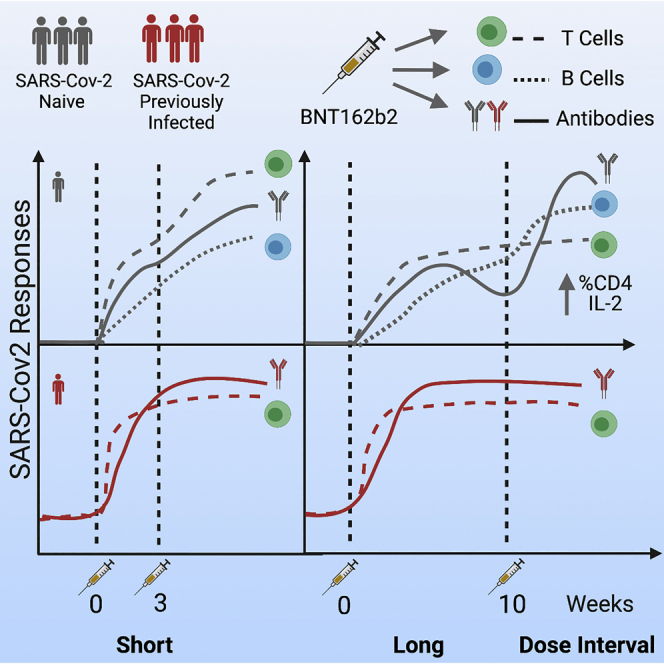

## Teaser

After giving a primary dose, delaying administration of a second dose of BNT162b2 COVID-19 vaccine up to 6–14 weeks continues to provide strong protection and contributes to favorable antibody, B cell, and T cell responses.

## Introduction

On December 31, 2020, the United Kingdom Chief Medical Officers announced changes to the dosing regimen for the second dose of the Pfizer/BioNTech BNT162b2 and Oxford/AstraZeneca severe acute respiratory syndrome coronavirus 2 (SARS-CoV-2) vaccines, with the interval between the first and second dose extended from 3–4 weeks to up to 12 weeks. This policy was implemented in a bid to avert deaths and prevent hospitalization because of severe coronavirus disease 2019 (COVID-19) and facilitated rapid rollout of one dose of the SARS-CoV-2 vaccine, providing a degree of cover as quickly as possible to prevent disease in a large proportion of higher-risk groups (Department of Health and Social Care UK, 2021).

The strategy to change regimens was based on estimates of efficacy after a single dose from clinical trials, modeling, and data from other vaccines. Although Pfizer’s clinical trial described an efficacy of 52% against symptomatic infection after a single dose (Polack et al., 2020), the United Kingdom’s Joint Committee on Vaccination and Immunisation (JCVI) estimated an efficacy of 89% against symptomatic infection, having removed infection data from within the first 14 days following the first dose (JCVI, 2020). However, this vaccine is based on novel mRNA technology, and little is known about the durability of immune responses after a single dose or the effect of extending dosing intervals.

The success of this strategy depends on the real-world effectiveness of the single dose of vaccine and on the effect of dose intervals on the boost. Viral variants such as delta (B.1.167.2) may also affect the protective efficacy of the generated immune responses (Reuters, 2021). We currently lack clear correlates of protection against SARS-CoV-2, although recent attempts to compare vaccines have given some indication of binding and neutralizing antibody measures that accompany efficacy (Earle et al., 2021; Feng et al., 2021; Khoury et al., 2021). Importantly, large-scale clinical trial data, on which these estimates are based, do not include measures of T cells. Because T cells are of increasing interest for providing protection (Rydyznski Moderbacher et al., 2020; Tan et al., 2021), human data to support such a role for cellular immunity are very valuable.

The United Kingdom SARS-CoV-2 Immunity & Reinfection Evaluation (SIREN) study is a multicenter prospective cohort study of staff in National Health Service (NHS) hospitals. Because of rapid rollout of vaccines to NHS workers in December 2020 and the high burden of infection with the alpha variant during the United Kingdom’s second wave, the SIREN study has emerged as a leading study of the real-world effectiveness of vaccines for SARS-CoV-2 (Hall et al., 2021). The PITCH (Protective Immunity from T cells in Healthcare Workers) study is a multicenter study nested within SIREN, focused on mechanistic studies, including T cell responses, of immunity to SARS-CoV-2 (Angyal et al., 2021). Samples from the PITCH study have helped define many features of the serologic response to variants following natural infection and different vaccine regimens (Dejnirattisai et al., 2021; Liu et al., 2021; Skelly et al., 2021; Supasa et al., 2021; Zhou et al., 2021) and confirmed robust immune responses following a single dose of vaccine in previously infected donors (Angyal et al., 2021). Shortly after initiating the United Kingdom vaccine program, the government announced a change in protocol for BNT162b2 with a dosing interval of up to 12 weeks. Only a fraction of staff received the conventional (short interval) dosing—the majority received extended interval dosing.

We aimed to track antibody (Ab) and T cell responses after the first dose of BNT162b2 mRNA vaccine and compare the magnitude of Ab and T cell responses 4 weeks after dose 2 between short and long vaccination regimens, coupling this with the protective efficacy data. We observed substantial clinical protection in the face of the alpha variant, accompanied by rapid induction of humoral and cellular immune responses, including neutralizing Abs (NAbs), most marked in those with a history of prior infection. Extension of the interval led to a higher level of NAbs following the second dose. These data define the effects of dose extension and offer immunological support for decision-making regarding vaccine dosing intervals.

## Results

### Protection induced by BNT162b2 using an extended dosing interval

**Figure 1:**
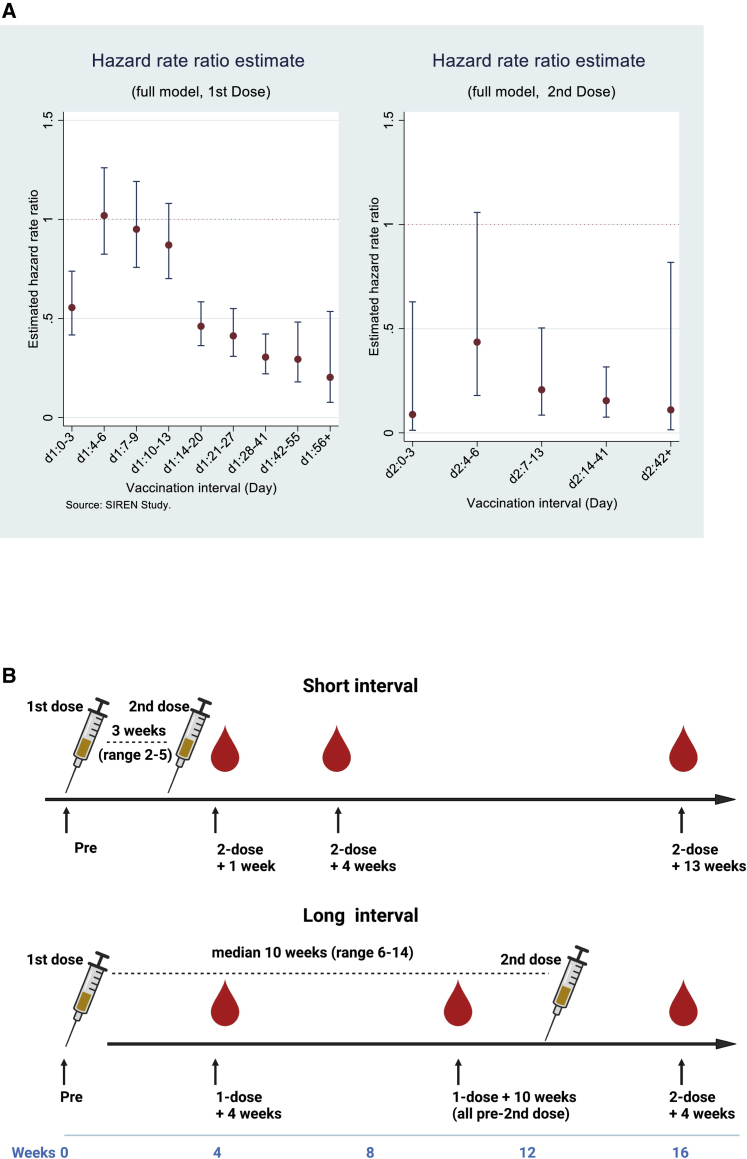
Vaccine efficacy and study design (A) Adjusted hazard ratios with 95% confidence intervals for PCR confirmed cases by interval after first and second doses of vaccination (source: SIREN study). HCWs underwent regular asymptomatic PCR screening (n = 25,066; negative cohort, 16,423; positive cohort, 8,643) with follow-up to 95 days after the first dose of the BNT162b2 vaccine. The hazard ratios are adjusted (including for age, ethnicity, comorbidities, and region), with full methodology described (Hall et al., 2021). (B) Schematic showing the dosing strategies of short and long vaccine intervals and phlebotomy time points.

To demonstrate the effect of extended dosing intervals on vaccine effectiveness against infection, we analyzed data from the entire SIREN study cohort. This study undertook clinical follow-up of 25,066 healthcare workers (HCWs) between December 7, 2020 and March 12, 2021 with asymptomatic screening by PCR over a period of up to 95 days (13.6 weeks) from the first dose of BNT162b2. At this time, the alpha (B.1.1.7) variant was the dominant circulating virus in the United Kingdom. These data are derived from prospective follow-up of the cohort (Hall et al., 2021). The time-resolved data show a gradual increase in the estimate of protection against all infections (asymptomatic and symptomatic) afforded by the vaccine following the first dose in individuals who were seronegative prior to vaccination (Figure 1A). A hazard ratio for infection of less than 50% is reached after about 14 days, with protection maintained at high levels until the second dose and then until the end of the follow-up period (although with wide confidence intervals because of decreasing numbers). The hazard ratios are adjusted (including for age, ethnicity, comorbidities, and region), and the lower hazard ratio seen on days 0–3 is explained by deferral of vaccination in symptomatic individuals. Overall, these data show robust protection against infection following the first dose of BNT162b2, with vaccine effectiveness reaching 72% by 3 weeks after dose 1 and maintained following the boosting second dose.

### Participants of the PITCH study

589 participants received two doses of the BNT162b2 Pfizer/BioNTech vaccine between December 9, 2020 and May 23, 2021 in 5 United Kingdom NHS hospital centers, with the majority undergoing the extended dosing schedule. The median age was 43 years (interquartile range [IQR], 32–52; range, 21–71) with 74% (431 of 582 reported) females, reflecting the demographics of the parent SIREN study (Hall et al., 2021), and 15% (72 of 482 reported) from an ethnic minority group (Table 1). Individuals were defined as SARS-CoV-2 naive (334, 57%) or previously infected (255, 43%) based on documented PCR and/or serology results from local NHS trusts or the mesoscale discovery (MSD) assay spike (S) and nucleocapsid (N) Ab results. In those infected previously, 59% (150 of 255) had a SARS-CoV-2-positive PCR test result a median of 8.7 (IQR, 7.5–9.3) months prior to vaccination.

**Table 1:** Characteristics of HCWs included in the study and dosing intervals

	All	Short (2–5 weeks)	Long (6–14 weeks)
**Dosing interval**			

Median days	70	23.5	71
Median weeks	10.00	3.36	10.14
IQR (days)	63–77	21–27	64–77
Range (days)	14–105	14–35	45–105
N	589	86	503
Female, n (%)	431 (74%)	45 (56%)	386 (77%)
Male, n (%)	151 (26%)	36 (44%)	115 (23%)
Sex unreported, n	7	5	2
Mean age	42.30	44.96	41.87
Age in years, median (IQR)	43 (32–52)	45 (37–54)	43 (31–51)
Age range	21–71	22–64	21–71

**Infection status**			

Naive, N (%)	334 (57%)	57 (66%)	277 (55%)
Previous SARS-CoV-2, N (%)	255 (43%)	29 (34%)	226 (45%)

**Ethnicity (self reported)**			

White, n (%)^a^	410 (85.1%)	58 (84%)	352 (85%)
Asian, n (%)^a^	45 (9.3%)	7 (10%)	38 (9%)
Black, n (%)^a^	7 (1.5%)	0 (0%)	7 (2%)
Other, n (%)^a^	20 (4.1%)	4 (6%)	16 (4%)
Unreported, n	107	17	90

IQR, interquartile range.

a Percentage of reported ethnicities.

The vaccine dosing interval was the conventional “short” 2- to 5-week interval (n = 86; median, 24 days; IQR, 21–27; range, 14–35) or a “long” 6- to 14-week interval (n = 503; median, 71 days; IQR, 64–77; range, 45–105) (Figure 1B; Table 1). An overview of the assays is shown in Table S1.

### Priming and boosting of serologic responses to SARS-CoV-2 using the extended dosing schedule

**Figure 2:**
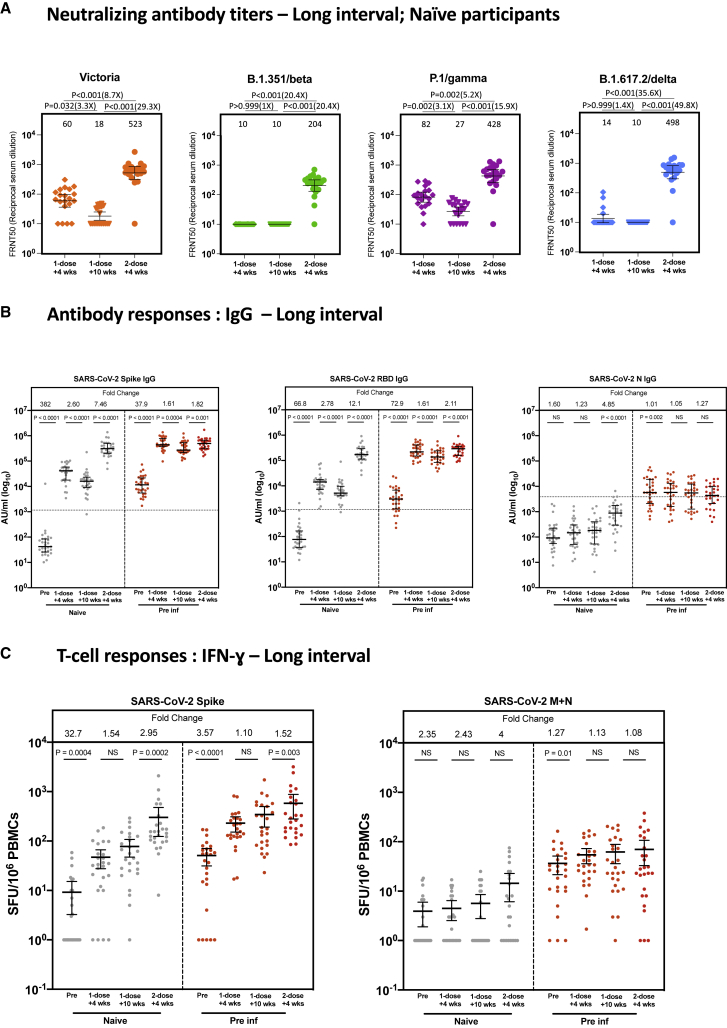
The long dosing interval with the BNT162b2 (Pfizer-BioNTech) vaccine elicits distinct NAb titer profiles against SARS-CoV-2 variants of concern and maintains T cell responses (A) NAbs against the Victoria isolate, B.1.351 (beta), P.1 (gamma), and B.1.617.2 (delta) taken from naive participants 4 (n = 20) and 10 weeks (n = 20) after the first vaccine dose and 4 weeks (n = 20) after the second vaccine dose in the long interval cohort. x axis, weeks since dose. Geometric mean neutralizing titers are shown immediately above each column and marked by a horizontal line on each column with 95% confience intervals. FRNT, focus reduction neutralization assay; FRNT_50_, the reciprocal dilution of the concentration of serum required to produce a 50% reduction in infectious focus-forming units of virus in Vero cells (ATCC, CCL-81). (B) SARS-CoV-2 spike (S)-, receptor binding domain (RBD)-, and nucleocapsid (N)-specific IgG time course using multiplexed MSD immunoassays in 29 naive and 29 pre-infected individuals vaccinated with a long interval between the doses. Data are shown in arbitrary units (AU)/mL. Horizontal dotted lines represent the cutoff of each assay based on pre-pandemic sera. (C) Comparison of IFNγ ELISpot responses to S (Victoria) from cryopreserved peripheral blood mononuclear cells (PBMCs) in 26 naive individuals and 26 previously infected individuals with a long interval between doses. Data are shown in spot-forming units per million PBMCs (SFU/10^6^). Gray circles, naive individuals; red circles, previously infected individuals. Pre, before vaccine; 1-dose + 4 weeks, 4 weeks after the first dose; 1-dose + 10 weeks, 8–12 weeks after the first dose; 2-dose + 4 weeks, 4 weeks after the second dose. Bars for (B) and (C) represent the median with interquartile range. Time points for (A) were compared with Kruskal-Wallis nonparametric test and Dunn’s multiple comparisons tests, with p values shown above linking lines and fold changes in brackets. Paired comparisons were performed for (B) and (C) using the Wilcoxon matched pairs signed rank test, with fold change values referring to the p value comparisons directly below. Data in (B) from 51 of the pre-vaccine and 51 of the 1-dose + 4 weeks responses as well as data from (C) from 51 of the pre-vaccine and 51 of the 1-dose + 4 weeks responses have been published previously (Angyal et al., 2021).

We next explored the immune responses accompanying this protection in the inter-dose interval. First, using SARS-CoV-2-naive individuals in the smaller immunology cohort (PITCH study), NAb levels were tested using a live virus microneutralization assay, as reported previously (Dejnirattisai et al., 2021; Liu et al., 2021; Supasa et al., 2021; Zhou et al., 2021). Measurable NAb titers against the early pandemic virus (Victoria) were observed in the majority of participants tested 4 weeks after the first dose (all infection naive), with a median 50% focus reduction neutralization titer (FRNT_50_) of around 10^2^ at this time point (Figure 2A). For the tested variant viruses—beta (B.1.351), gamma (P.1), and delta (B.1.167.2)—there was very limited detection of NAbs against beta and delta after one dose but only a minimal reduction in titers against gamma compared with Victoria. Although the alpha variant was not assayed in this set of experiments, extensive previous comparisons have indicated a consistent drop in titer of around 3-fold (Supasa et al., 2021). These titers declined up to 3-fold following the peak and were boosted markedly following the second dose.

**Figure S1:**
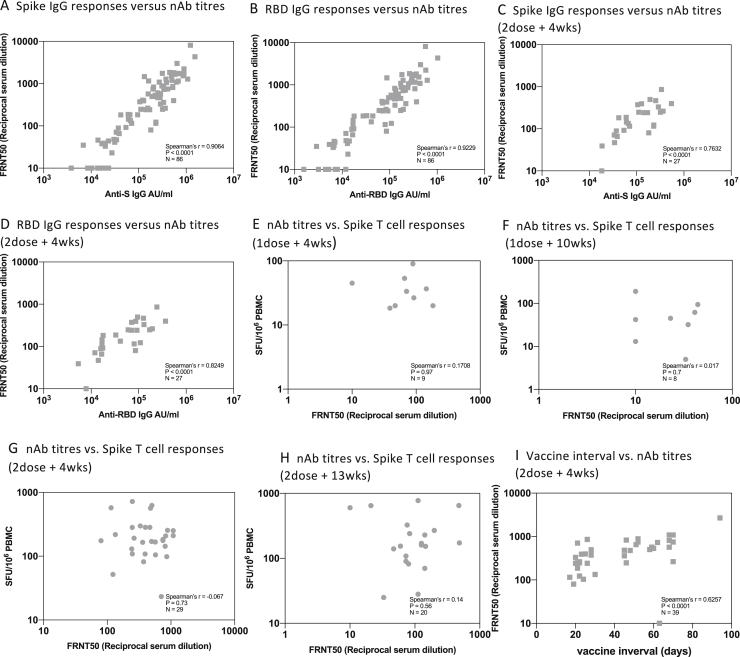
Correlation of NAb titers with T cell and IgG responses and vaccine interval, related to Figures 2 and 3A A. Relationship between IgG response to Spike (MSD) and neutralizing antibody (nAB) response to Victoria from all time points and vaccine dosing intervals . B. Relationship between IgG response to Receptor binding domain (MSD) and nAB response to Victoria from all time points and vaccine dosing intervals. C. Relationship between IgG response to spike (MSD) and nAB response to Victoria at dose-2 plus 4 weeks in short (3 weeks) and long (10 weeks) vaccine dosing intervals. D. Relationship between IgG response to Receptor binding domain (MSD) and nAB response to Victoria at dose-2 plus 4 weeks weeks in short (3 weeks) and long (10 weeks) vaccine dosing intervals. E. Relationship between Nab to Victoria and T cell responses to Spike at dose-1 plus 4 weeks. F. Relationship between Nab to Victoria and T cell responses to Spike at dose-1 plus 10 weeks. G. Relationship between Nab to Victoria and T cell responses to Spike at dose-2 plus 4 weeks weeks in short (3 weeks) and long (10 weeks) vaccine dosing intervals. H. Relationship between Nab to Victoria and T cell responses to Spike at dose-2 plus 13 weeks in short (3 weeks) and long (10 weeks) vaccine dosing intervals I. Correlation between vaccine dosing interval and neutralizing antibodies at 2nd dose plus 4 weeks. Spearman’s correlation was performed. Grey symbols indicate naive participants.

Using a multiplex ELISA (MSD) to measure anti-S Abs, we saw a similar pattern of decline following the first vaccine dose and boosting following the second dose in naive participants (Figure 2B). A parallel phenomenon was seen in the previously infected group; the levels of anti-S Ab prior to the first dose were present at a level approaching that seen after a single dose in the naive group and boosted substantially by the first dose. Overall, neutralization levels correlated with S and receptor binding domain (RBD) binding titers (Figures S1A–S1D). Linear mixed-effects regression models were used to confirm these findings after adjusting for age and sex (Table 2).

**Table 2:** Linear mixed-effects regression models of T cell (IFNγ) or Ab (IgG) immune responses

	S T cell responses in naive participants			S T cell responses in pre inf participants			S Ab responses in naive participants			S Ab responses in pre inf participants				
**Coefficient**	**Estimates**	**CI (95%)**	**p Value**	**Estimates**	**CI (95%)**	**p Value**	**Estimates**	**CI (95%)**	**p Value**	**Estimates**		**CI (95%)**		**p Value**

Intercept	1.47	0.95–1.98	<0.001^a^	1.92	1.26–2.59	<0.001^a^	4.84	4.42–5.26	<0.001^a^	5.77		5.41–6.12		<0.001^a^
Age	−0.00	−0.02 to 0.01	0.705	0.01	−0.01 to 0.02	0.480	−0.01	−0.02 to 0.00	0.090	−0.00		−0.01 to 0.01		0.640
Sex (M)	−0.15	−0.54 to 0.23	0.435	0.42	0.01–0.84	0.046^a^	0.09	−0.26 to 0.45	0.608	−0.04		0.29 to 0.21		0.757
Time point (pre vaccine)	−0.79	−1.08 to −0.50	<0.001^a^	−0.88	−1.09 to −0.67	<0.001^a^	−2.79	−2.95 to −2.63	<0.001^a^	−1.65		−1.77 to −1.54		<0.001^a^
Time point (dose 1 + 10 weeks)	0.28	−0.01 to 0.57	0.060	0.08	−0.13 to 0.28	0.466	−0.38	−0.53 to −0.22	< 0.001^a^	−0.18		−0.29 to −0.07		0.002^a^
Time point (dose 2 + 4 weeks)	0.85	0.56–1.13	<0.001^a^	0.31	0.10–0.51	0.003^a^	0.97	0.81–1.12	<0.001^a^	−0.02		−0.13 to 0.10		0.793

**Random effects**														

σ^2^	0.28			0.14			0.09			0.05				
τ_00_	0.06 _PubID_			0.12 _PubID_			0.09 _PubID_			0.04 _PubID_				
ICC	0.18			0.46			0.49			0.43				
N	26 _PubID_			26 _PubID_			29 _PubID_			29 _PubID_				
Observations	103			104			116			116				
Marginal R^2^/conditional R^2^	0.506/0.593			0.462/0.710			0.914/0.956			0.848/0.913				

Shown are four linear mixed-effect regression models (LMERs) of T cell or Ab responses in naive or pre-inf individuals across vaccine time points in the long vaccine dose regimen. Variables include age, sex, and time point. Variable references are sex (F [female] versus M [male]), time point (dose 1 + 4 weeks versus pre-vaccine/dose 1 + 10 weeks/dose 2 + 4 weeks.

a Indicates statistical significance.

In summary, a clear humoral response against the vaccine strain virus is induced by a single dose of vaccine across the cohort, although with NAbs at relatively low levels, especially against the beta variant and delta variants. The peak NAb response following priming is followed by a decline during the extended dose interval, most marked in naive individuals, with a boost after the second dose.

### Induction and maintenance of anti-S T cell responses using the extended dosing schedule

We next investigated the T cell response using an established interferon-gamma (IFNγ) enzyme-linked immune absorbent spot (ELISpot) assay (Angyal et al., 2021; Ogbe et al., 2021). The S-specific T cell response was well maintained during the 10 weeks following the primary vaccine, with no evidence of contraction, a phenomenon that was seen equally in the previously infected and naive groups (Figure 2C). T cells were boosted by the second dose in both groups, with the greatest effect seen in the naive group. In contrast, responses to the non-S T cell targets (membrane [M] and N) showed only minimal changes over this period in both groups (Figure 2C). Linear mixed-effects regression models were again performed to confirm these results after adjusting for age and sex (Table 2).

These data demonstrate maintenance of cellular responses using the extended dosing approach. We did not see a significant relationship between NAbs and the T cell response to S (Figures S1E–S1H).

### Extension of the dosing interval leads to an increase in peak NAbs and B cells but not T cells

**Figure 3:**
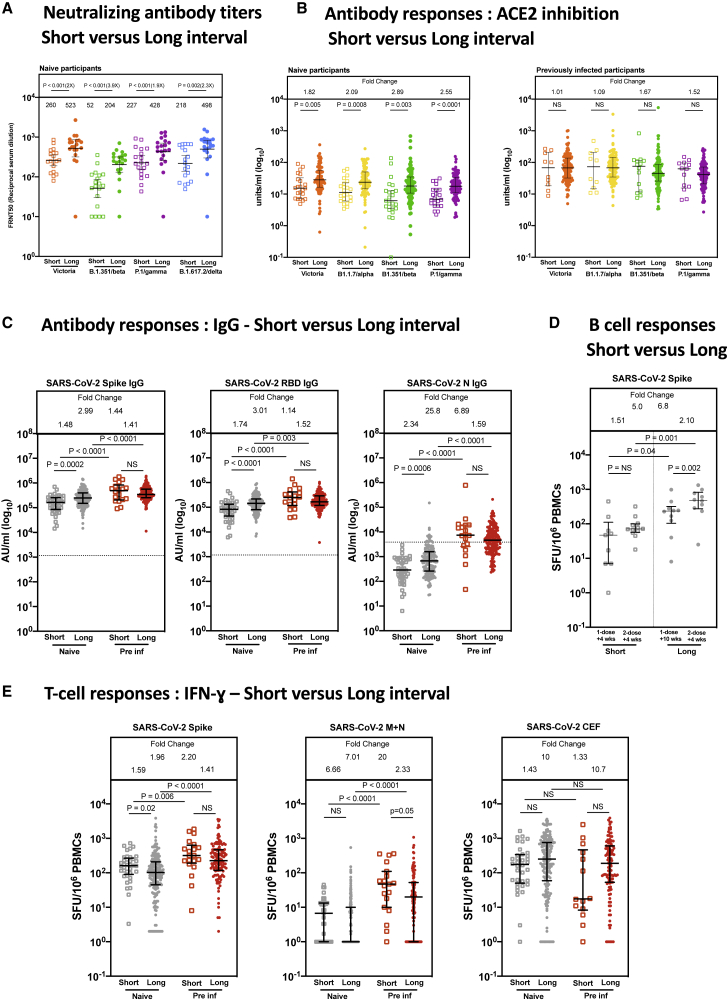
Comparison of IgG responses and T cell responses 4 weeks after the second dose of vaccine (A) Comparison of NAbs against the Victoria isolate, B.1.351 (beta), P.1 (gamma), and B.1.617.2 (delta) 4 weeks after the second dose with short (n = 19) and long (n = 20) interval in naive participants. There is a median of 3.3 weeks (range, 2.4–4.3) between doses in the short interval cohort and 8.4 weeks (range 6.4-10) in the long interval cohort. Geometric mean neutralizing titers with 95% confidence intervals are shown. FRNT, focus reduction neutralization assay; FRNT_50_, the reciprocal dilution of the concentration of serum required to produce a 50% reduction in infectious focus-forming units of virus in Vero cells (ATCC, CCL-81). (B) Effect of a short and a long vaccine dosing interval on the ability of sera to inhibit ACE2 binding to SARS-CoV-2 S (Victoria, B.1.1.7 [alpha], B.1.351 [beta], or P.1 [gamma]) 4 weeks after the second dose. ACE2 inhibition was analyzed using a multiplexed MSD assay. Data are shown in units/mL. Bars represent the median with 95% confidence intervals. Naive, short: n = 23; naive, long: n = 94; previously infected (pre inf), short: n = 14; pre inf, long: n = 119. (C) Effect of a short or a long vaccine dosing interval on SARS-CoV-2 S-, RBD-, and N-specific IgG responses in naive (gray circles) and pre individuals (red circles). IgG responses were measured in serum 4 weeks after the second dose using multiplexed MSD immunoassays and are shown in arbitrary units (AU)/mL. Naive, short: n = 41; naive, long: n = 151; pre inf, short: n = 19; pre inf, long: n = 169. Horizontal dotted lines represent the cutoff of each assay based on pre-pandemic sera. (D) IgG B ELISpot responses from cryopreserved peripheral blood mononuclear cells (PBMCs) 4 weeks after the first dose in naive short (n = 9), 2 weeks after the second dose in naive short (n = 12), 10 weeks after the first dose in naive long (n = 10), and 2 weeks after the second dose in naive long (n = 10). Values are expressed as spot-forming units per million PBMCs (SFU/10^6^) representing anti-S IgG-secreting cells. (E) IFNγ ELISpot responses from cryopreserved PBMCs 4 weeks after the second dose in naive short (n = 37), naive long (n = 188), pre inf short (n = 20), and pre inf long (n = 124) individuals. Values are expressed as SFU/10^6^. Displayed are responses to peptide pools representing S1 and S2 subunits of S (Victoria), peptide pools representing membrane (M) and N proteins (N) and cytomegalovirus, Epstein-Barr virus, influenza, and tetanus antigens (CEF). Bars represent the median with interquartile range for (B), (C), (D), and (E). Time points were compared with two-tailed Mann-Whitney tests, with p values shown above linking lines and fold changes in brackets for (A) and fold change values referring to the p value comparisons directly below for (B)–(E).

We next compared immune responses in the SARS-CoV-2-naive cohort vaccinated using the longer dosing interval with those vaccinated using the conventional 3- to 4-week (short) interval. We noted higher NAb titers (4 weeks after the second dose of both regimens, all infection naive) in individuals vaccinated using the long interval regimen, with a 2- to 4-fold increase in titer, depending on the variant tested (Figure 3A), with a correlation across time (Figure S1I). In each case, titers against the Victoria (B) virus were greater than against the tested beta, gamma, and delta variants, with the greatest reduction in titer noted against the beta variant, where the benefit of the longer dosing interval was also greatest. These data were confirmed using a secondary assay based on RBD binding inhibition to ACE2 (MSD) (Figure 3B). Again, a clear increase is seen with extended dosing across the tested variants (including the alpha variant, which was circulating widely during the period of this study), and this was most evident in the infection-naive cohort.

We also tested the effect of the dosing interval on binding Abs using S, RBD, and N as targets, splitting the cohort by previous exposure. A clear advantage of the longer interval was seen again, although only in the naive cohort (Figure 3C). The previously infected cohort had equally high levels of binding to S and RBD regardless of vaccine regimen (Figure 3C). Generalized linear regression models were performed to confirm these findings after adjustment for age, sex, and previous infection status, with separate models run for naive and previously infected individuals (Table 3). No effect of ethnicity was seen in a reduced dataset (n = 143) where this information was available (Table S2).

**Table 3:** Generalized linear regression models of T cell (IFNγ) or antibody (IgG) immune responses

	S T cell responses in naive and pre inf participants at dose 2 + 4 weeks			S Ab responses in naive participants at dose 2 + 4 weeks			S Ab responses in pre inf participants at dose 2 + 4 weeks		
**Coefficient**	**Estimates**	**CI (95%)**	**p Value**	**Estimates**	**CI (95%)**	**p Value**	**Estimates**	**CI (95%)**	**p Value**

Intercept	2.21	1.90–2.51	<0.001^a^	5.40	5.18–5.63	<0.001^a^	5.50	5.26–5.73	<0.001^a^
Age	−0.00	−0.01 to 0.00	0.522	−0.01	−0.01 to −0.00	0.020^a^	0.00	−0.00 to 0.00	0.554
Sex (M)	−0.06	−0.21 to 0.08	0.404	−0.10	−0.20 to 0.00	0.060	0.02	−0.09 to 0.13	0.690
Pre inf (yes)	0.38	0.24–0.51	<0.001^a^						
Vaccine dose interval (long)	−0.19	−0.37 to −0.01	0.041^a^	0.20	0.08–0.32	0.001^a^	0.01	−0.14 to 0.17	0.886
Observations (R^2^)	374 (0.086)			189 (0.131)			184 (0.003)		

Shown are three generalized linear models (GLMs) of T cell (naive and pre inf individuals), Ab (naive individuals), and Ab (pre inf individuals) responses 4 weeks after the second dose. Variables include age, sex, previous infection, and vaccine dose interval. Variable references are sex (F [female] versus M [male]), previous infection (yes versus no), and vaccine dose interval (short versus long). CI, confidence interval.

a Indicates statistical significance.

**Figure S2:**
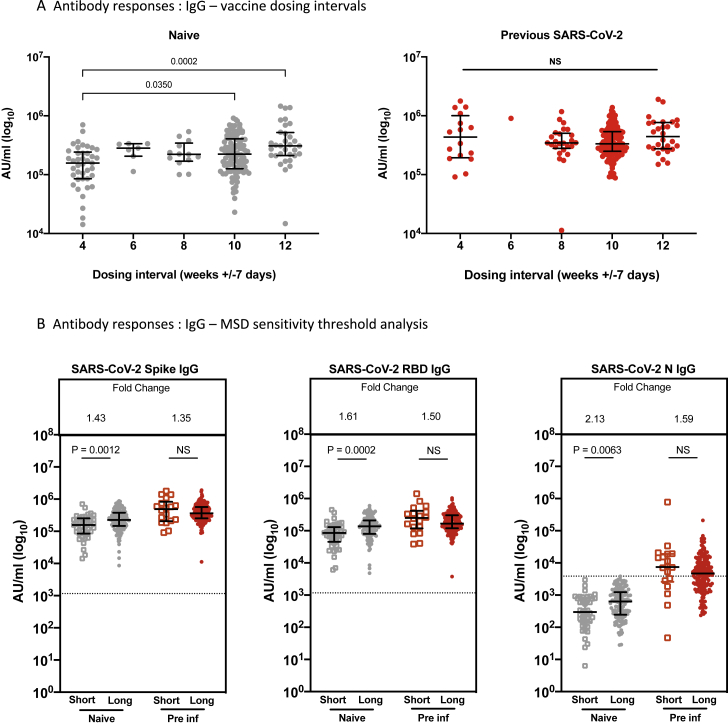
Effect of vaccine dosing interval and MSD sensitivity threshold on IgG responses, related to Figures 2B and 3C A. Effect of a dosing interval grouped 4 weekly on SARS-CoV-2 S-specific IgG responses in naive (gray symbols) and pre-infected individuals (red symbols). IgG responses were measured in serum 4weeks after the second dose using multiplexed MSD immunoassays and are shown in Arbitrary Units/ml (AU/ml). Bars represent the median with interquartile range. Unpaired comparisons between the groups were performed using a Mann-Whitney test. B. Effect of a short or a long vaccine dosing interval on SARS-CoV-2 S-, RBD- and N-specific IgG responses in naive (gray symbols) and pre-infected individuals (red symbols) after removing participants with IgG N responses above the sensitivity threshold (3,874 AU/ml). IgG responses were measured in serum 4 weeks following the second dose using multiplexed MSD immunoassays and are shown in Arbitrary Units/ml (AU/ml). Bars represent the median with interquartile range. Unpaired comparisons between the groups were performed using a Mann-Whitney test. Horizontal dotted lines represent the sensitivity threshold of each assay based on pre-pandemic sera.

Comparing anti-S responses with dosing interval grouped around 4-, 6-, 8-, 10-, and 12-week intervals, we saw a significant difference for infection-naive HCWs between dose intervals of 4 versus 10 or 12 weeks but no significant differences between other intervals, such as 8 weeks versus 12 weeks, and no difference for previously infected HCWs (Figure S2A). A number of individuals defined previously as naive at baseline on the basis of serology and infection history (14 of 138, 10.1%) showed reactivity to N in this MSD immunoglobulin G (IgG) assay 4 weeks after the second dose, suggesting infection/exposure during the period of observation. We tested whether removing these individuals in the naive group who seroconvert to anti-N affected the result (Figure S2B) and found that the higher binding Ab responses to S and RBD remained significant.

SARS-CoV-2 S-specific B cell responses showed induction and maintenance during the extended interval between the first and second dose in infection-naive participants (Figure 3D), and, indeed, the magnitude of the antigen-specific B cell response 10 weeks after one dose in the extended interval cohort was higher than 4 weeks after one dose in the short interval cohort, supporting continued B cell development beyond 4 weeks after a prime. 4 weeks after the second dose, we saw a nearly 7-fold increase in the magnitude of the B cell response for the extended interval cohort compared with the short interval, in parallel to the higher Abs seen.

**Figure S3:**
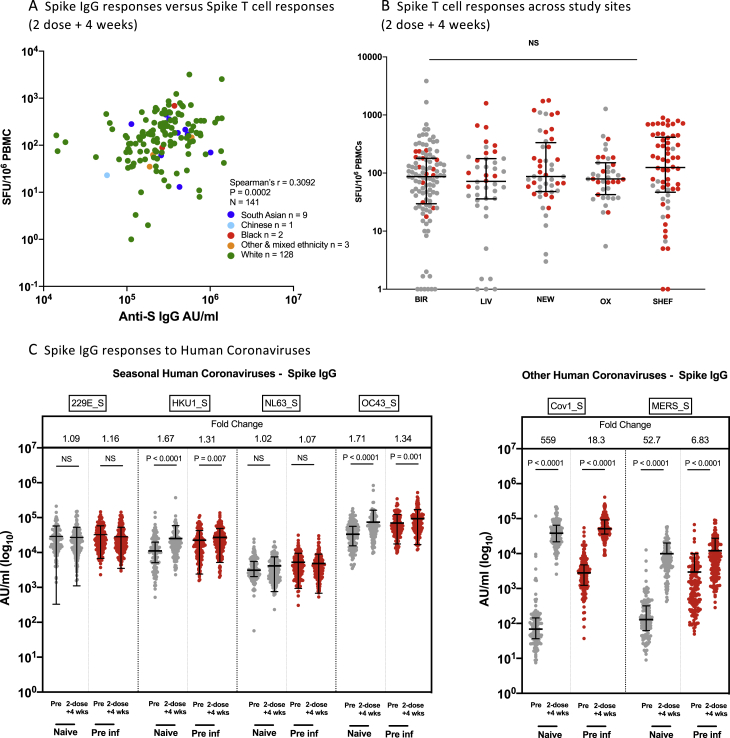
Effect of ethnicity and study site on IgG and T cells responses and comparison of IgG responses with alphacoronavirses and betacoronaviruses 4 weeks after the second dose of vaccine, related to Table 2 and Figure 3 A. Correlation of IFN-y ELISpot responses to Spike B and anti-spike IgG response, in participants 4 weeks after the second dose in naive, and previously infected individuals who received either the long or short interval dose. Data points are colored by ethnic group. Spearman’s correlation was performed. B. Comparison of of IFN-y ELISpot responses to Spike B, from cryo-preserved PBMCs 4 weeks after second dose in naive, and previously infected individuals who received the long interval dose, across the 5 centers (BIR: Birmingham, LIV: Liverpool, NEW: Newcastle, OX: Oxford, SHEF: Sheffield). Data are shown as spot-forming units per million peripheral blood mononuclear cells (SFU/10^6^ PBMCs). Unpaired comparisons across two groups were performed using the Mann Whitney test. Grey symbols represent naive individuals, Red symbols represent previously infected individuals. C. Alpha coronavirus and beta coronavirus spike-specific IgG responses in naive (gray circles, n = 151) and pre-infected individuals (red circles, n = 169) vaccinated with a long interval between the doses. IgG responses were measured in unpaired sera before vaccination (pre) and4 weeks after the second dose (1-dose +4 wks) using multiplexed MSD immunoassays. Data are shown in Arbitrary Units/ml (AU/ml). Bars represent the median with interquartile range. Unpaired comparisons between the groups were performed using a Mann-Whitney test. Fold change values each refer to the P value comparisons directly below.

Extension of the dosing interval did not lead to greater induction of T cell responses following the second dose (Figure 3E). Indeed, although for those infected previously there was no difference detected, for those previously naive we found a modestly lower T cell response weeks after the longer dosing interval compared with the shorter interval. Responses to control antigens (CEF - cytomegalovirus, Epstein-Barr virus, and influenza) were unaffected by prior exposure or regimen, whereas responses to SARS-CoV-2 M and N proteins were, as expected, associated with prior exposure but stable over time. There was a weak correlation between binding Abs and T cell response 4 weeks after the second dose, with no obvious effect of reported ethnicity (Figure S3A) although the study was underpowered for full evaluation of ethnicity. Generalized linear regression models were performed to confirm these findings after adjustment for age, sex, and previous infection status (Table 3). No effect of ethnicity was seen in a reduced dataset where this information was available (n = 277; Table S2).

**Figure 4:**
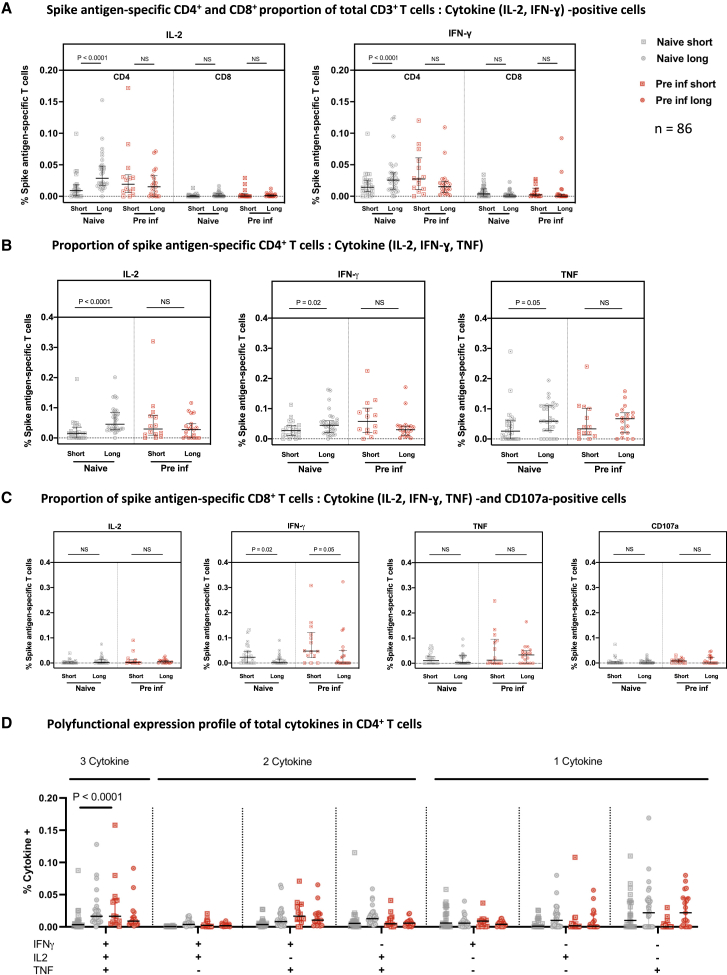
Analysis of S-specific T cell responses by flow cytometry Cryopreserved PBMCs from 86 participants who received the short or long vaccine dosing interval, with S antigen-specific ELISpot responses over 40 SFU/million PBMCs 4 weeks after the second dose, were analyzed by ICS and flow cytometry. (A) The T cell populations responsible for IFNγ or IL-2 expression were assessed by reporting the ratio of IFNγ- or IL-2-expressing cells among CD4^+^ or CD8^+^ cells, expressed as a proportion of their CD3^+^ live population. (B and C) The individual cytokine expression levels of total IFNγ, IL-2, or TNF are reported as a proportion of the (B) CD4^+^ T cell population or (C) CD8^+^ T cells with addition of CD107a (a marker of cytotoxicity). (D) Polyfunctionality was evaluated by expression of one or more cytokines in CD4^+^ cells, showing the number of cytokines released in each group and against each IFNγ, IL-2, and/or TNF gated combination shown as a proportion of total CD4^+^ T cells. Naive short, n = 23; naive long, n = 30; pre inf short, n = 14; pre inf long, n = 19. Bars represent the median with interquartile range. Unpaired comparisons across two groups were performed using Mann-Whitney test, and paired comparisons were performed using Wilcoxon matched pairs signed rank test. Grey circles, naive individuals; red circles, pre inf individuals.

**Figure S4:**
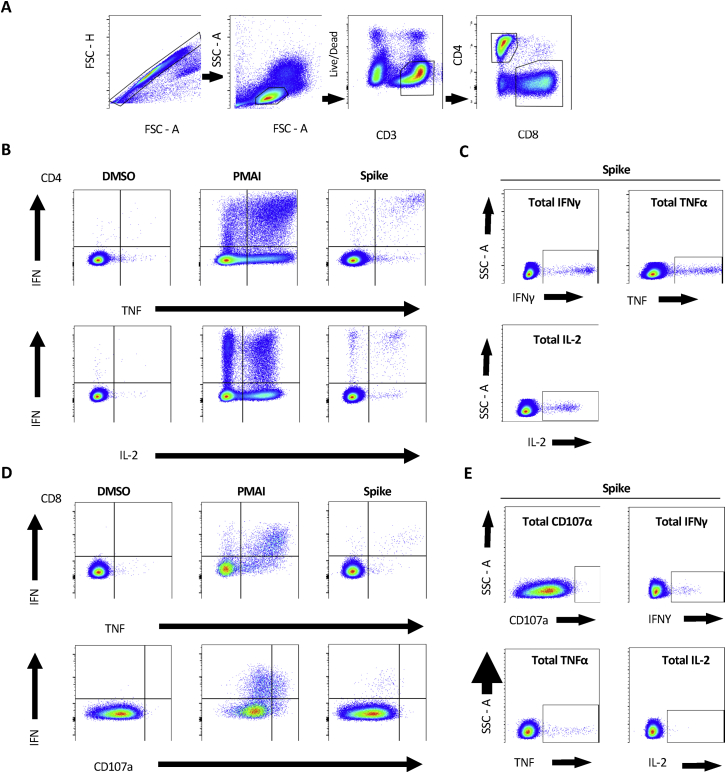
Representative gating strategy for ICS analysis, related to Figure 4 (A) Description of gates left-to-right showing sequential gates used in the initial analysis of each subject. Cell doublets were removed using forward scatter (FSC) parameters. Lymphocytes were selected by FSC and side scatter (SSC), CD3 positive but non-Live-dead stained living populations were carried forward to isolate CD4+ and CD8+ T Cells. (B) The gates used to define differences between different cytokine expression and (C) total individual cytokine expression in CD4+ and (D,E) CD8+ respectively. DMSO and PMAI represent negative and positive control conditions to demonstrate the contrast in cytokine expression. These were also used in setting the gate boundaries in each subject for the analysis of SARSCoV2 Spike protein responses.

We next compared the functionality of these T cell responses 4 weeks after the second dose in more depth in 86 HCWs using intracellular cytokine staining (ICS). For this analysis, we selected only participants with positive ELISpot assays, which we defined as > 40 spot-forming units per million peripheral blood mononuclear cells (PBMCs), the mean of the DMSO negative values + 2 standard deviations, because, in previous studies (Angyal et al., 2021; Ogbe et al., 2021), we observed that the smaller frequency populations are hard to detect by flow cytometry and also prone to inaccuracy because of low cell numbers. We saw marked skewing in the CD3^+^ T cell compartment toward a S-specific CD4^+^ T cell response with the long dosing interval but more balance between CD4^+^ and CD8^+^ for the short interval (Figure 4A; representative gating strategy in Figure S4). Further analysis showed that, within the CD4^+^ compartment, S-specific responses for all cytokines tested were higher in naive participants using the long dosing interval compared with the short interval, whereas no differences were observed in the previously infected participants (Figure 4B). In contrast, within the CD8^+^ compartment, there were no differences in S-specific responses except for CD8^+^ IFNγ responses, which were lower in participants on the long dosing interval, irrespective of pre-infection status (Figure 4C).

These experiments revealed that infection-naive recipients of the long dosing interval generated a higher interleukin-2 (IL-2) CD4^+^ response to S compared with the short dosing interval, along with higher IFNγ and tumor necrosis factor (TNF) CD4^+^ responses (Figure 4B), whereas the CD8^+^ response was reversed, with lower IFNγ responses for the long interval (Figure 4C).

By comparing the proportions of polyfunctional CD4^+^ T cells between the long and short dosing groups, we observed an increased CD4^+^ T cell polyfunctionality in naive participants who had undergone the extended dosing interval compared with the short dosing interval (Figure 4D). No difference in polyfunctionality was observed in previously infected participants. The comparison of CD8^+^ T cell polyfunctionality was limited by the small number of HCWs who showed CD8^+^ responses sufficient for this analysis but did not suggest any large differences between the groups.

The combined ELISpot and ICS data show a modest difference in T cell responses between long and short regimens, with both regimens inducing and maintaining robust CD4^+^ and CD8^+^ responses in naive and previously infected donors. The longer dosing interval was linked to induction of higher levels of IL-2-secreting CD4^+^ T cells in the naive group, accompanied by slightly lower levels of IFNγ-secreting CD8^+^ T cells.

### Comparison of naive and previously infected individuals

**Figure S5:**
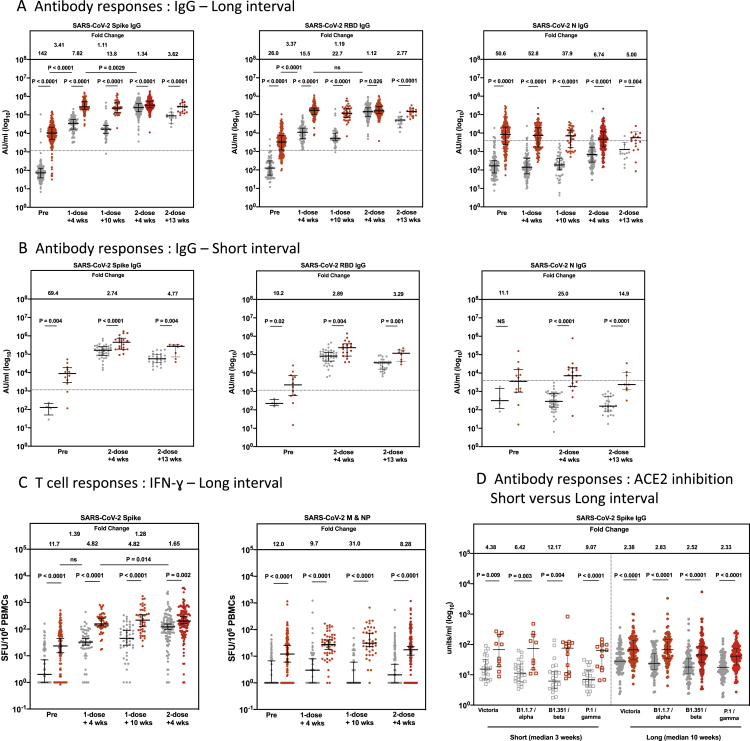
Effect of previous SARS-CoV-2 infection on magnitude of IgG and T cell responses, related to Figure 2 A-B. SARS-CoV-2 spike(S)-, receptor-binding domain (RBD)- and nucleocapsid(N)-specific IgG time course in naive (gray circles, n = 234) and pre-infected individuals vaccinated (red circles, n = 228) with a long (A) or short (B) interval between the doses. IgG responses were measured in unpaired sera before vaccination (pre), 4 weeks after the first dose (1-dose +4 wks), 8-12 weeks after 1^st^ dose (1-dose + 10 wks), 4 weeks after the second dose (2-dose + 4wks), and 13 weeks after the second dose (2-dose +13 wks) using multiplexed MSD immunoassays. Data are shown in Arbitrary Units/ml (AU/ml). C. Comparison of IFN-y ELISpot responses to Spike (Victoria) from cryo-preserved peripheral blood mononuclear cells (PBMCs) in 276 naive individuals and 165 previously-infected individuals unmatched for pre-vaccine (pre), 4 weeks after 1st dose (1-dose +4 wks), 8-12 weeks after 1st dose (1-dose +10 wks) and 4 weeks after 2nd dose (2-dose +4 wks). Data are shown as spot-forming units per million PBMCs (SFU/10^6^ PBMCs). D. Impact of a short and a long vaccine dosing interval on the ability of sera to inhibit ACE2 binding to SARS-CoV-2 spike (Victoria, B.1.1.7 (alpha), B.1.351 (beta) or P.1 (gamma)) 28 days after the second dose. ACE2 inhibition was analyzed using a multiplexed MSD assay. Data are shown in units/ml. Naive, short: n = 23; Naive, long: n = 94; Pre inf, short: n = 14; Pre inf, long: n = 119. Bars represent the median with interquartile range. Unaired comparisons between the groups were performed using a Mann-Whitney test. Fold change values each refer to the P value comparisons directly below. Horizontal dotted lines represent the cut-offs of each assay based on pre-pandemic sera. Data from 51 of the pre-vaccine, and 51 of the 1-dose +4 wks responses were previously published (Angyal et al., 2021).

Throughout the analyses, we noted differences in responses between naive and previously infected individuals. To explore this further, we used a larger dataset of all available unpaired data across the time course (n = 589 participants) and performed a side-by-side analysis of humoral and cellular responses following first and second dose, including 13 weeks (3 months) after the second dose where samples were available (Figures S5A–S5D). 4 weeks after a single dose of vaccination, previous infection gives a significant advantage in the magnitude of anti-S IgG Abs (approximately 8--fold; Figure S5A) and of T cell response (approximately 5-fold; Figure S5C) (as reported in Angyal et al., 2021). 4 weeks after 2 doses, there is still a statistically significant difference in Ab and T cell response between those with and without previous infection, although the magnitude is less pronounced (up to 3-fold). In those with previous infection, there was consistent evidence of higher neutralization activity against variants of concern using the RBD binding inhibition to ACE2 assay (Figure S5D), suggesting that “hybrid immunity” from previous infection plus vaccination gives the strongest cross-reactive neutralization.

We recently identified an association between the interval separating prior infection until the first dose and the magnitude of Ab response to the first dose in previously infected individuals. This association shows that a longer interval is associated with a stronger IgG response to first dose of the vaccine (Angyal et al., 2021). Our finding that higher IgG levels are associated with longer dosing intervals in naive individuals provides parallel evidence that longer intervals between antigen exposure favors induction of higher Ab levels.

Two doses of BNT162b2 induced Ab against the S protein of other betacoronaviruses, with novel induction of IgG against severe acute respiratory syndrome (SARS) and Middle East respiratory syndrome (MERS) viruses (Figure S3C) and boosting of baseline IgG against the human seasonal betacoronaviruses HKU1 and OC43. There was no significant boosting of baseline Abs against the human seasonal alphacoronaviruses NL63 and 229E.

### Dynamics of immune responses following boosting

**Figure 5:**
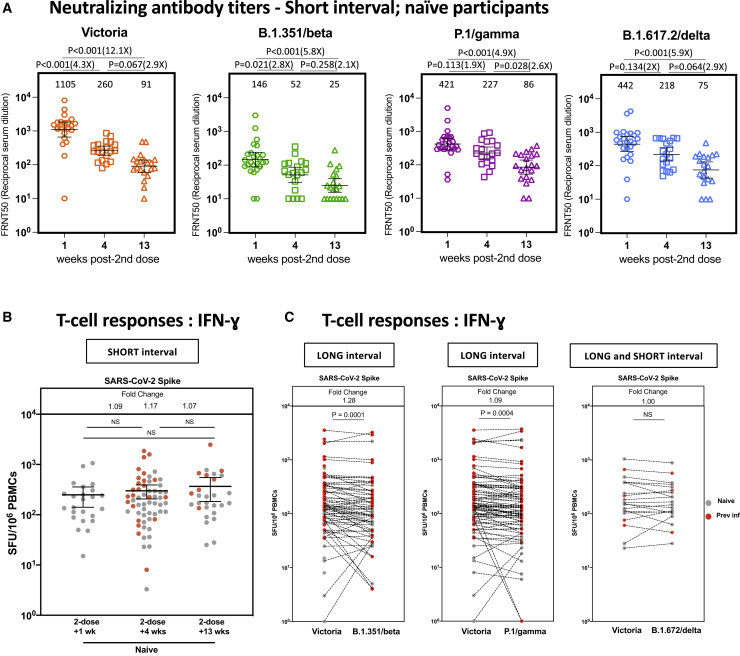
Short dosing interval with the BNT162b2 (Pfizer-BioNTech) vaccine elicits distinct NAb titer profiles against SARS-CoV-2 variants of concern that decline over time, whereas T cell responses are maintained (A) NAb titers, measured by FRNT, against the early pandemic Victoria isolate, B.1.351 (beta), P.1 (gamma), and B.1.617.2 (delta) from naive participants in weeks 1 (n = 25), 4 (n = 19), and 13 (n = 20) after the second vaccine in the short interval dose cohort (median dose interval, 3.3 weeks; range, 2.4–4.3). The x axis indicates weeks since dose. Geometric mean neutralizing titers with 95% confidence intervals are shown. Neutralization titers from dose 2 + 1 week (Victoria, B.1.351, P.1, and B.1.617.2) and dose 1 + 4 weeks and 10 weeks (Victoria and B.1.617.2) have been reported previously (Dejnirattisai et al., 2021; Liu et al., 2021; Supasa et al., 2021; Zhou et al., 2021). (B) Comparison of IFNγ ELISpot responses to S Victoria from cryopreserved PBMCs in 43 naive individuals and 26 pre inf individuals who received the short interval dose 1, 4, and 13 weeks after the second dose. (C) Comparison of IFNγ ELISpot responses from cryopreserved peripheral blood mononucelar cells (PBMCs) in 40 naive individuals and 42 pre inf individuals matched for responses to S Victoria, spike B.1.35/beta, and S P.1/gamma. Individuals received the long interval dosing regimen, and samples were taken 4 weeks after the second dose. Data are shown as spot forming units (SFU) per million PBMCs. Time points for (A) were compared with Kruskal-Wallis non-parametric test and Dunn’s multiple comparisons tests. The p values are illustrated above linking lines and fold changes in brackets. Bars represent the median with interquartile range for (B) and (C). Time points were compared with two-tailed Mann-Whitney tests for (A) and (B), and paired comparisons were performed using Wilcoxon matched pairs signed rank test for (C), with fold change values referring to the p value comparisons directly below. Gray circles, naive individuals; red circles, pre inf individuals.

We next addressed the dynamics of immune responses following a second dose boost. Tracking NAb responses following the short regimen, we found a peak 1 week after the second dose, with a subsequent clear decline in circulating NAb titers against Victoria and variant viruses over the subsequent time points (4 weeks and 13 weeks after the second dose, all infection naive; Figure 5A). The decline against the Victoria strain between weeks 1 and 4 is approximately 4-fold, which compares with around 3-fold between weeks 4 and 13 and, thus, around 12-fold over the overall 3-month period. Future studies will evaluate the durability of the extended dosing interval.

We observed maintenance of T cell responses over the same period (Figure 5B). These responses showed a very modest but statistically significant loss of activity against peptide pools from the variant S protein sequences—namely, the beta (1.18-fold lower, p = 0.0001) and gamma (1.1-fold lower, p = 0.0016) variants compared with the wild-type sequence—but no loss of activity against the delta variant (fold change = 1.00, p = 0.2058) Figure 5C).

These data recapitulate the data seen after a single dose. That is, there is a clear decline in circulating Abs over a 3-month period, with strong maintenance of the T cell response. This is a period well studied in clinical trials, where robust protection following a second dose was observed (Polack et al., 2020).

## Discussion

Extended dose intervals for boosting COVID-19 mRNA vaccines were introduced based on an interpretation of clinical trial data for BNT162b2 and extrapolation from the AstraZeneca vaccine and vaccines for other pathogens but without a strong dataset for BNT162b2 to support the immunology or real-world clinical effectiveness. Here we provide an extensive serological and T cell assay dataset to explore the vaccine effectiveness seen in the parent SIREN study. The serologic response to one or two doses of BNT162b2 falls over time and is higher after an extended dosing interval in infection-naive participants compared with the 3- to 4-week dosing interval that was tested in the licensing trials. As reported previously (Dan et al., 2021; Goel et al., 2021; Turner et al., 2021), we found S-specific B cell responses to be well maintained in contrast to the Ab responses, and we found them to be around 7-fold higher in the infection-naive extended dosing interval cohort. The T cell response is well maintained after one and two doses. The sustained T cell response from 1 to 10 weeks after the first dose in the extended dosing interval group contrasts with the waning of T cells we and others (Dan et al., 2021; Tomic et al., 2021) have reported after natural infection. In addition, maintenance of T cell responses from 1 to 13 weeks after the second dose contrasts with some other longitudinal vaccine studies where T cell responses peaked 1 week after boost (Esposito et al., 2020; Swadling et al., 2014; Venkatraman et al., 2019). The T cell response is of a marginally lower magnitude after the longer dosing interval when measured by the ELISpot assay of T cell effector function, with higher IL-2, IFNγ, and TNF CD4^+^ responses and lower IFNγ CD8^+^ responses compared with the 3- to 4-week dosing interval. Ongoing work when this cohort reaches 6 months after the second dose will evaluate whether the variation in T cell functionality 4 weeks after the second dose translates into differences in sustained memory.

The data from the larger clinical study in SIREN provide clear evidence of a protective effect of a single dose against the circulating alpha variant over an extended period. The SIREN study is based on prospective screening for infection rather than reactive testing based on symptoms alone and therefore provides a robust measurement of protection. The number of unvaccinated individuals dropped over the period of study, leading to a wide confidence interval for the estimates of efficacy, but there is no evidence of a decline over the first 3 months and, if anything, a trend to increasing protection, which is sustained following the boost.

Protection over this extended interval between doses and, indeed, after the boost may be provided by a range of mediators, including circulating NAbs and effector T cells as we have measured, but also many factors beyond peripheral blood that we have not measured. There is likely to be a strong component of protection mediated by mucosal immune responses, including local IgA and IgG (Fröberg and Diavatopoulos, 2021), as well as resident memory T cells in the respiratory tract (Niessl et al., 2021), which requires further investigation in future. Several studies have demonstrated functional Ab responses beyond neutralizing function against SARS-CoV-2 after infection and vaccination (Barrett et al., 2021; Bartsch et al., 2021; Tomic et al., 2021). Other interesting observations that support a role of T cells in protection come from analyses in the setting of the beta variant outbreak in South Africa (Moore et al., 2021). T cell responses are not substantially affected overall by the emerging variants, in contrast to NAbs, which show poor cross-reactivity, in particular with the beta variant. The protective efficacy of vaccines such as Ad26 in South African trials has therefore been hypothesized to link to cellular immunity. Other data that have explored the relative role of Ab and T cells in protection come from more mechanistic animal studies, where depletion of CD8^+^ T cells in SARS-CoV-2 convalescent macaques affected protective natural immunity against re-challenge when Ab levels were waning (McMahan et al., 2021).

The most significant observation of this study is that boosting after a longer interval leads to maintained immunogenicity. There is a distinct effect on anti-S responses, with an increase in NAbs, as seen for longer dosing intervals with the AstraZeneca 1222 vaccine (Voysey et al., 2021), but a modest reduction in the IFNγ-producing ELISpot response compared with conventional (short) interval dosing. Although the difference in T cell response is quite small, it is reproducible with different assays and was also seen in a study of an elderly United Kingdom cohort (Parry et al., 2021). The short and long dosing regimens result in induction of substantial T cell responses. The short dosing interval gives a slightly higher IFNγ-producing T cell response, consistent with effector functions, with CD4^+^ and CD8^+^ contributions, whereas the longer dosing interval results in a CD4^+^ dominated phenotype with marked IL-2 production to S. A study from Public Health England (PHE) has confirmed higher Ab levels after extended dosing intervals compared with short ones and has provided the first evidence of high vaccine effectiveness for extended dosing interval (Amirthalingam et al., 2021). As the authors discuss, some residual confounding between groups is likely to remain, and further studies will follow. There is currently no agreed correlate of protection, but it has been suggested that, in a study of AZD122, an average level of 40,923 arbitrary units (a.u.)/mL 4 weeks after the second dose for anti-S IgG using the MSD assay used in this study, equivalent to 264 binding Ab units (BAUs)/mL using the World Health Organization (WHO) international standard, is associated with 80% protection from symptomatic infection for the population of recipients receiving that vaccine regimen (Feng et al., 2021). However, this was predominantly against the alpha variant. The average levels for all groups in our study exceeded this threshold, in line with other studies reporting higher Ab levels after double vaccination with BNT162b2 compared with AZD122 (Liu et al., 2021).

The CD4^+^ helper T cell response induced by the extended dosing interval gives potential mechanistic insight into the increased Ab boost because IL-2 provides important help for B cells to develop into plasmablasts (Hipp et al., 2017; Le Gallou et al., 2012). Benefits of longer intervals between vaccine doses have been observed for other vaccines in mouse and human studies, where early boosting results in higher numbers of terminally differentiated and effector T cells, whereas later boosting promotes efficient T cell expansion and enhanced long-term memory cell persistence (Capone et al., 2020; Steffensen et al., 2013). Therefore, a longer interval between vaccine doses in infection-naive people may allow S-specific T cells to fully differentiate into memory T cells that respond optimally to S re-exposure. Interestingly, extension of the interval between prior infection and prime in a group with previous exposure also leads to enhanced humoral immunity (Angyal et al., 2021). Because the first vaccine in previously infected individuals is effectively a boost for the memory pools concerned, this is consistent with the current study’s observation of higher humoral immunity for extended dosing interval recipients in the naive group and the observation that there is much less of an effect of dosing interval for the previously infected group.

Our study builds on findings from studies showing that, after a single vaccine dose, previous infection primes for higher Ab and T cell responses after vaccination (Angyal et al., 2021; Bradley et al., 2021; Ebinger et al., 2021; Manisty et al., 2021). After two doses of vaccine, previous infection continues to give an advantage in terms of the measured Ab and T cell response and cross-neutralization to variants of concern, albeit with a less marked difference in magnitude compared with after a single dose. The functional importance of this in terms of protection and effect on durability of the responses remain to be elucidated. Past infection may confer other advantages, including mucosal immunity, Ab and T cell responses to other viral antigens beyond S, and differences in the character of the response.

Regardless of the dosing regimen, there remains a large amount of inter-individual variability in the vaccine responsiveness for humoral and cellular responses. Overall, we see a correlation between the two measures when responses across all time points are considered, but there was no relationship between NAb and T cell responses (ELISpot) when the time point 4 weeks after the second dose was considered alone. The effect of the dosing interval and the effect of boosting on individuals with prior infection was much less evident than in naive individuals. We also examined the influence of age, sex, and ethnicity in our cohort. Although these observations are limited by the numbers studied and balance of the cohort, we did not observe any substantial effect in simple comparative or multivariate analyses except for a modest effect of older age associated with lower Ab levels in naive HCWs. It remains likely, however, that genetic effects, such as human leukocyte antigen (HLA) type, do play a role because this has been shown in other vaccine settings (Mentzer et al., 2015), and the effect of exposure to other betacoronaviruses remains to be explored further.

The immunogenicity of longer regimens appears to be robust and, for Ab measurements, improved over the conventional 3- to 4-week regimen. We provide evidence that T cells are induced and sustained during the longer period between doses in the 6- to 14-week regimen, but there is an effect of dosing interval on the relative proportion of T cell subsets. Ongoing studies in this cohort will monitor the durability of Ab and T cell responses 6 months after a second 2^nd^ vaccine dose delivered in an extended dosing interval and response to third “booster” doses where given. For policy makers, optimal dosing intervals may depend on community prevalence, population immunity from natural infection, circulating variants of concern, and vaccine supply. A short dosing interval gives early protection, whereas an increased interval appears to improve peak NAb levels.

### Limitations of the study

This study provides detailed information at relative scale about the immune response to the BNT162b2 vaccine in a healthy, working-age population to help us understand vaccine effectiveness against the alpha variant seen in the SIREN study and in the United Kingdom as a whole. The limitations of this study include first of all the predominance of females and white ethnicity, reflective of the HCWs we were able to recruit, although neither female sex nor ethnicity were revealed as significant variables in our modeling analysis. Second, laboratory measurements such as low NAb levels are subject to threshold effects and may not reflect true functional immunity upon re-exposure to the S protein. Third, ongoing follow-up to evaluate the effect of dosing interval on the durability of the immune response is needed. Fourth, the high heterogeneity of Ab and T cell responses we observed means that our findings of higher levels of Abs and T cell memory function after an extended dosing interval are of most relevance at a population level rather than at an individual level. Fifth, although we know the vaccine effectiveness in the parent cohort (SIREN), we did not measure this directly in the same individuals in whom we made T cell and Ab measurements, meaning that we cannot directly measure novel correlates of protection in this study. Finally, the group sizes were skewed toward a much bigger number of participants in the long interval schedule because the sudden change in policy during the rollout, at the time of peak pressure from COVID-19 hospitalizations on the United Kingdom health service, meant that we were limited regarding the number of individuals receiving the short interval schedule we could recruit. On the other hand, this rapid change in policy gave us a unique opportunity to directly compare the immunogenicity of the extended versus the standard dosing interval.

The importance of immune memory and the multiple ways in which the immune system after vaccination can prevent severe disease, including the role of memory pools, and specific functional properties of binding Abs and cellular responses must be borne in mind to avoid excessive focus on point measures of circulating NAb levels as a singular proxy for vaccine-induced immunity. Our study demonstrates that two doses of BNT162b2 are highly immunogenic for Abs and T cells across the studied range of dosing intervals. When community levels of circulating SARS-CoV-2 virus are low, the extended dosing interval appears to be suitable for immunogenicity, but this needs to be weighed against the more immediate benefits of two doses over one. Policy decisions around vaccine dosing intervals will depend on several factors, including the current prevalence of SARS-CoV-2, which variants of concern are emerging, population susceptibility, and vaccine supply. However, robust immunologic data can now inform such policies.

## STAR★Methods

### Key resources table

**Table:** 

REAGENT or RESOURCE	SOURCE	IDENTIFIER
**Antibodies**		

Purified NA/LE mouse anti-human CD28 (Clone: CD28.2)	BD Biosciences, UK	Cat#555725; RRID:AB_396068
Purified NA/LE mouse anti-human CD49d (Clone: 9F10)	BD Biosciences, UK	Cat#555501; RRID:AB_2130052
BV510 mouse anti-human CD3 (Clone: UCHT1)	BD Biosciences, UK	Cat#563109: RRID:AB_2732053
Brilliant Violet 421 mouse anti-human CD8 (Clone: RPA-T8)	BD Biosciences, UK	Cat#562428; RRID:AB_11154035
PE mouse anti-human TNFα (Clone: MAb11)	Thermo Fisher Scientific,	Cat#12-7349-82; RRID:AB_466208
PERCP/ Cyanine 5.5 mouse anti-human CD4 (Clone: RPA-T4)	Biologend, UK	Cat#300530: RRID:AB_394493
APC-Cy7 mouse anti-human CD14 (Clone: MφP9)	BD Biosciences, UK	Cat#557831: RRID:AB_396889
FITC mouse anti-human IFNγ Clone: 45-15)	Miltenyi Biotec Ltd	Cat#130-113-492; RRID:AB_2733589
APC mouse anti-human IL-2 (5344.111)	BD Biosciences, UK	Cat#341116; RRID:AB_400574
PE-Cy7 mouse anti-human CD107α (Clone: H4A3)	BD Biosciences, UK	Cat#561348; RRID:AB_10644018
Human FluoroSPOT anti-IgG capture mAb	Mabtech	Cat#FSX-05R-10
Human anti-NP (mAb206)	Dejnirattisai et al., 2021	N/A
Anti-Human IgG (Fc specific)-Peroxidase	Sigma	Cat#A0170; RRID:AB_257868

**Bacterial and virus strains**		

SARS-CoV-2/P.1	Dejnirattisai et al., 2021	N/A
SARS-CoV-2/B.1.617.2	Wendy Barclay and Thushan De Silva	N/A
SARS-CoV-2 (Australia/VIC01/2020)	Caly et al., 2020	N/A
SARS-CoV-2/B.1.351	Public Health England	N/A

**Chemicals, peptides, and recombinant proteins**		

TrueBlue Peroxidase Substrate	Insight Biotechnology	Cat#5510-0030
Dulbecco’s Modified Eagle Medium, high glucose	Sigma-Aldrich	Cat#D5796
Custom synthesized peptides (18-mers)	Mimotopes	http://www.mimotopes.com
Dimethyl Sulfoxide	Sigma	Cat#D2650-100ML
RPMI-1640 Medium with Sodium bicarbonate, no L-Glutamine	Sigma	Cat#R0883
L-Glutamine	Sigma	Cat#G7513
Penicillin/Streptomycin	Sigma	Cat#P0781
Fetal Bovine Serum	Sigma	Cat#F9665-500ML
Lymphoprep	StemCell Technology	Cat#07861
L-Glutamine–Penicillin–Streptomycin solution	Sigma-Aldrich	Cat#G1146
GlutaMAX Supplement	GIBCO	Cat#35050061
Phosphate buffered saline tablets	Fisher Scientific	Cat#12821680
Fetal Bovine Serum	GIBCO	Cat#12676029
Carbonate/bicarbonate capsules	Sigma Aldrich	Cat#C3041-100CAP
ProMix CEF peptide pool	Proimmune, Oxford	Cat#PX-CEF
Phytohemagglutinin-L	Sigma Aldrich	Cat#11249738001
Carboxymethyl cellulose	Sigma	Cat#C4888
Tween 20	Sigma Aldrich	Cat#P2287-500ml
1-Step NBT/BCIP Substrate Solution	Life Technologies	Cat#34042
LIVE/DEAD fixable near-IR dead cell stain kit	Thermo Fisher Scientific	Cat#L34975
Perm/Wash Buffer (10x)	BD Biosciences	Cat#554723
Brefeldin A	Merck, UK	Cat#B6542
PMA	Merck, UK	Cat#P1585
Ionomycin calcium salt from *Streptomyces conglobatus*	Merck, UK	Cat#I0634
DPBS, no calcium, no magnesium	Thermo Fisher Scientific	Cat#14190144
37% Formaldehyde solution	Merck, UK	Cat#F8775
GIBCO Fetal Bovine Serum, qualified, heat inactivated	Thermo Fisher Scientific, UK	Cat#10500064
RPMI-1640 Medium with sodium biicarbonate but without L-Glutamine	Merck, UK	Cat#R0883
Bovine Serum Albumin (BSA)	Merck, UK	Cat#A9418

**Critical commercial assays**		

V-PLEX COVID-19 Coronavirus Panel 3 (IgG) Kit	Meso Scale Discovery, Rockville, MD USA	Cat#K15399U-2
V-PLEX SARS-CoV-2 Panel 7 (ACE2) Kit	Meso Scale Discovery, Rockville, MD, USA	Cat#K15440u
Human IgA/IgG FluoroSpotFLEX kit	Mabtech	Cat#X-06G05R-10
Human memory B cell stimpack	Mabtech	Cat#3660-1
Human IFNγ ELISpot Basic kit	Mabtech	Cat#3420-2A

**Deposited data**		

Immunogenicity of standard and extended dosing intervals of BNT162b2 mRNA vaccine. Payne et al.	Mendeley Data	https://doi.org/10.17632/fyp26zjgmj.1

**Experimental models: Cell lines**		

Vero cells	ATCC	Cat#CCL-81

**Software and algorithms**		

Discovery Bench 4.0	Meso Scale Discovery, Rockville, MD, USA	Immunoassay Analysis Software | Meso Scale Discovery
Prism 8.0	GraphPad	https://www.graphpad.com/scientific-software/prism/
IBM SPSS Software 26	IBM	https://www.ibm.com/us-en/?ar=1
AID ELISpot software 8.0	Autoimmun Diagnostika	http://www.elispot.com/products/software
Flojo 10.7.1	BD Biosciences	https://www.flowjo.com/
R version 4.0.4 (2021-02-15)–“Lost Library Book”	Web-based open source software	https://www.r-project.org
R studio version 1.1.463	Web-based open source software	https://www.rstudio.com

**Other**		

FacsCanto II cytometer	BD Biosciences UK	N/A

### Resource availability

#### Lead contact

• Further information and requests for resources and reagents should be directed to and will be fulfilled by the lead contact, Paul Klenerman (paul.klenerman@ndm.ox.ac.uk).

#### Materials availability

• This study did not generate new unique reagents.

### Experimental model and subject details

#### Primary Cell cultures

Vero cells (ATCC CCL-81) were cultured in Dulbecco’s Modified Eagle Medium, high glucose (Sigma-Aldrich), supplemented with 10% (v/v) heat-inactivated fetal bovine serum (GIBCO), 100 units/ml penicillin, 100 μg/ml streptomycin, 2mM L-Glutamine (Sigma) and 2mM GlutaMax (GIBCO).

#### Viral Stocks

Live virus experiments were conducted in containment level 3 facilities in line with UK’s Advisory Committee on Dangerous Pathogens (ACDP) guidelines. SARS-CoV-2/human/AUS/VIC01/2020 (Caly et al., 2020), SARS-CoV-2/B.1.1.7 and SARS-CoV-2/B.1.351 were provided by Public Health England, P.1 from a throat swab from Brazil were grown in Vero (ATCC CCL-81) cells. Cells were infected with the SARS-CoV-2 virus using an MOI of 0.0001. Virus containing supernatant was harvested at 80% cytopathic effect, and spun at 3000 rpm at 4°C before storage at −80°C. Viral titers were determined by a focus-forming assay on Vero cells. Victoria passage 5, B.1.1.7 passage 2, B.1.351 passage 4 and P.1 passage 1 stocks were sequenced to verify that they contained the expected spike protein sequence and no changes to the furin cleavage sites. The B.1.617.2 virus was kindly provided Wendy Barclay and Thushan De Silva and contained the following mutations compared to the Wuhan sequence: T19R, G142D, Δ156-157/R158G, A222V, L452R, T478K, D614G, P681R, D950N.

#### Human participants

##### The PITCH study

Healthcare workers (HCWs) were recruited to the PITCH study Consortium Study at National Health Service (NHS) hospitals in five centers in England (University Hospitals Birmingham NHS Foundation Trust, Liverpool University Hospitals NHS Foundation Trust, Newcastle upon Tyne Hospitals NHS Foundation Trust, Oxford University Hospitals NHS Foundation Trust, and Sheffield Teaching Hospitals NHS Foundation Trust). Participants were recruited both with and without a history of SARS-CoV-2 infection. Individuals were defined as SARS-CoV-2 naive or previously infected based on documented PCR and/or serology results from local NHS trusts, or the MSD assay S and N antibody results if these data were not available locally. All participants received the BNT162b2 Pfizer/BioNTech vaccine. The vaccine dosing interval was either a “short” 3-5 week interval (median 24 days, IQR 21-27, range 14-35), or a “long” 6-14 week interval (median 71 days, IQR 64-77, range 45-105). The immune response data from baseline and 4 weeks after the first dose has been previously reported, alongside data for 21 HCWs 4 weeks after the second vaccine in a short dosing regimen (Angyal et al., 2021). PITCH is a sub-study of the SIREN study which was approved by the Berkshire Research Ethics Committee, Health Research 250 Authority (IRAS ID 284460, REC reference 20/SC/0230), with PITCH recognized as a sub-study on 2 December 2020. SIREN is registered with ISRCTN (Trial ID:252 ISRCTN11041050). Some participants were recruited under aligned study protocols. In Birmingham participants were recruited under the Determining the immune response to SARS-CoV-2 infection in convalescent health care workers (COCO) study (IRAS ID: 282525). In Liverpool some participants were recruited under the “Human immune responses to acute virus infections” Study (16/NW/0170), approved by North West - Liverpool Central Research Ethics Committee on 8 March 2016, and amended on 14th September 2020 and 4th May 2021. In Oxford, participants were recruited under the GI Biobank Study 16/YH/0247, approved by the research ethics committee (REC) at Yorkshire & The Humber - Sheffield Research Ethics Committee on 29 July 2016, which has been amended for this purpose on 8 June 2020. In Sheffield, participants were recruited under the Observational Biobanking study STHObs (18/YH/0441), which was amended for this study on 10 September 2020. The study was conducted in compliance with all relevant ethical regulations for work with human participants, and according to the principles of the Declaration of Helsinki (2008) and the International Conference on Harmonization (ICH) Good Clinical Practice (GCP) guidelines. 589 participants were recruited in PITCH study; Female:Male ratio 74:26, Median age 43 (range 21-71). Further demographics are reported in Table 1. Written informed consent was obtained for all participants enrolled in the study.

##### The SIREN Study

The SIREN study is a separate ongoing study with the methodology published previously (Hall et al., 2021). SIREN is a large prospective cohort study of healthcare workers and allied staff aged 18 years and above working in UK National Health Service (publicly funded) hospitals. The vaccine effectiveness analysis included in Figure 1A of this paper presents a repeat analysis after an extended follow-up period (up to 12 March 2021) to that previously published (Angyal et al., 2021); data up to 5 February 2021. In brief, effectiveness of the BNT162b2 vaccine against PCR-confirmed infection (asymptomatic and symptomatic) was estimated in SIREN participants followed up from 7 December 2020 to 12 March 2012, by comparing time to infection in vaccinated and unvaccinated participants. Participants underwent fortnightly asymptomatic SARS-CoV-2 PCR testing and monthly antibody testing, and all tests (including symptomatic testing) outside SIREN were captured. Baseline risk factors were collected at enrolment, symptom status was collected every 2 weeks, and vaccination status was collected through linkage to the National Immunisations Management System and questionnaires. Historic SARS-CoV-2 PCR and antibody testing data was used to determine each participants prior SARS-CoV-2 infection status (positive or negative cohort) at the beginning of the analysis period (7 December 2020). Vaccine effectiveness of the BNT162b2 vaccine was calculated using a piecewise exponential hazard mixed-effects model (shared frailty-type model) using a Poisson distribution, which adjusted for the variable incidence during the follow-up period and important confounders. The study is registered with ISRCTN, number ISRCTN11041050, and is ongoing. 25,661 participants were included in this analysis, Female:Male ratio 84:16.

### Method details

#### Study design and sample collection

In this prospective, observational, cohort study, HCWs were recruited into the PITCH study from across the five centers. Individuals consenting to participate were recruited by word of mouth, hospital e-mail communications and from hospital-based staff screening programmes for SARS-CoV-2, including HCWs enrolled in the national SIREN study at three sites (Liverpool, Newcastle and Sheffield). Eligible participants were adults aged 18 or over currently working as an HCW, including allied support and laboratory staff, and were sampled for the current study between 4 December 2020 and 27 May 2021. Participants on the “long” dosing interval received phlebotomy for assessment of immune responses prior to first dose of vaccine (median 23 days, IQR 6/55), 4 weeks after the first dose (median 28 days, IQR 26/31), 8 weeks after the first dose (median 70 days, IQR 62/75), 4 weeks after the second dose (median 28 days, IQR 25/32) and 13 weeks after the second dose (median 94 days, IQR 91/103). Participants on the “short” dosing interval received phlebotomy for assessment of immune responses 1 week after the second dose (median 7 days, IQR 7/8,) 4 weeks after the second dose (median 28 days, IQR 27/33) and 13 weeks after the second dose (median 98 days, IQR 93/122). An overview of assays performed is detailed in Table S1. Clinical information including BNT162b2 immunisation dates, date of any prior SARS-CoV-2 infection defined by a positive PCR test and/or detection of antibodies to spike or nucleocapsid protein, presence or absence of symptoms, time between symptom onset and sampling, age, gender and ethnicity of participant was recorded.

#### Focus Reduction Neutralization Assay (FRNT)

The neutralization potential of antibodies (Ab) was measured using a Focus Reduction Neutralization Test (FRNT), where the reduction in the number of the infected foci is compared to a negative control well without antibody. Briefly, serially diluted Ab or plasma was mixed with SARS-CoV-2 strain Victoria or P.1 and incubated for 1 hr at 37°C. The mixtures were then transferred to 96**-**well, cell culture-treated, flat-bottom microplates containing confluent Vero cell monolayers in duplicate and incubated for a further 2 hr followed by the addition of 1.5% semi-solid carboxymethyl cellulose (Sigma) overlay medium to each well to limit virus diffusion. A focus forming assay was then performed by staining Vero cells with human anti-nucleocapsid monoclonal Ab (mAb206) followed by peroxidase-conjugated goat anti-human IgG (A0170; Sigma). Finally, the foci (infected cells) approximately 100 per well in the absence of antibodies, were visualized by adding TrueBlue Peroxidase Substrate (Insight Biotechnology). Virus-infected cell foci were counted on the classic AID ELISpot reader using AID ELISpot software. The percentage of focus reduction was calculated and IC_50_ was determined using the probit program from the SPSS package.

#### Mesoscale Discovery (MSD) binding assays

IgG responses to SARS-CoV-2, SARS-CoV-1, MERS-CoV and seasonal coronaviruses were measured using a multiplexed MSD immunoassay: The V-PLEX COVID-19 Coronavirus Panel 3 (IgG) Kit from Meso Scale Diagnostics, Rockville, MD USA. A MULTI-SPOT® 96-well, 10 spot plate was coated with three SARS CoV-2 antigens (S, RBD, N), SARS-CoV-1 and MERS-CoV spike trimers, as well as spike proteins from seasonal human coronaviruses, HCoV-OC43, HCoV-HKU1, HCoV-229E and HCoV-NL63, and bovine serum albumin. Antigens were spotted at 200−400 μg/mL (MSD® Coronavirus Plate 3). Multiplex MSD assays were performed as per the instructions of the manufacturer. To measure IgG antibodies, 96-well plates were blocked with MSD Blocker A for 30 minutes. Following washing with washing buffer, samples diluted 1:1,000-10,000 in diluent buffer, or MSD standard or undiluted internal MSD controls, were added to the wells. After 2-hour incubation and a washing step, detection antibody (MSD SULFO-TAG Anti-Human IgG Antibody, 1/200) was added. Following washing, MSD GOLD Read Buffer B was added and plates were read using a MESO® SECTOR S 600 Reader. The standard curve was established by fitting the signals from the standard using a 4-parameter logistic model. Concentrations of samples were determined from the electrochemiluminescence signals by back-fitting to the standard curve and multiplied by the dilution factor. Concentrations are expressed in Arbitrary Units/ml (AU/ml). Cut-offs were determined for each SARS-CoV-2 antigen (S, RBD and N) based on the concentrations measured in 103 pre-pandemic sera + 3 Standard Deviation. Cut-off for S: 1160 AU/ml; cut-off for RBD: 1169 AU/ml; cut-off for N: 3874 AU/ml.

#### MSD ACE2 inhibition assay

The V-PLEX SARS-CoV-2 Panel 7 (ACE2) Kit, from MSD, Rockville, MD, a multiplexed MSD immunoassay, was also used to measure the ability of human sera to inhibit ACE2 binding to SARS-CoV-2 spike (B, B.1, B.1.1.7, B.1.351 or P.1). A MULTI-SPOT® 96-well, 10 spot plate was coated with five SARS-CoV-2 spike antigens (B, B.1, B.1.1.7, B.1.351 or P.1). Multiplex MSD Assays were performed as per manufacturer’s instructions. To measure ACE2 inhibition, 96-well plates were blocked with MSD Blocker for 30 minutes. Plates were then washed in MSD washing buffer, and samples were diluted 1:10 – 1:100 in diluent buffer. Importantly, an ACE2 calibration curve which consists of a monoclonal antibody with equivalent activity against spike variants was used to interpolate results as arbitrary units (units/ml), with 1 unit being equivalent to 1ug/ml neutralising activity of the standard. Furthermore, internal controls and the WHO SARS-CoV-2 Immunoglobulin international standard (20/136) were added to each plate. After 1-hour incubation recombinant human ACE2-SULFO-TAG was added to all wells. After a further 1-hour plates were washed and MSD GOLD Read Buffer B was added, plates were then immediately read using a MESO® SECTOR S 600 Reader.

#### Memory B cell Fluorospot assay

Cryopreserved PBMCs were thawed and cultured for 72 hours with polyclonal stimulation containing 1μg/ml R848 and 10ng/ml IL-2 from the Human memory B cell stimpack (Mabtech). Using the Human IgA/IgG FluoroSpotFLEX kit (Mabtech), stimulated PBMCs were then added at 2x10^5^ cells/well to fluorospot plates coated with 10μg/ml Sars-CoV-2 spike glycoprotein diluted in PBS. Plates were incubated for 16 hours at 37°C and developed according to the manufacturer’s instructions (Mabtech). Analysis was carried out with AID ELISpot software 8.0 (Autoimmun Diagnostika). All samples were tested in triplicates and response was measured as spike-specific spots per million PBMCs with PBS background subtracted.

#### T cell ELISpot assays

The PITCH ELISpot Standard Operating Procedure has been published previously (Angyal et al., 2021). Interferon-gamma (IFNγ) ELISpot assays were set up from cryopreserved PBMCs using the Human IFNγ ELISpot Basic kit (Mabtech 3420-2A). A single protocol was agreed across the centers as previously published (Angyal et al., 2021), and we found no significant difference in magnitude of ELISpot response to spike 4 weeks after the second vaccine across the five centers (Figure S3B).

MultiScreen-IP filter plates (Millipore, MAIPS4510) were coated with 50ul per well using the ELISpot Basic kit capture antibody (clone 1-D1K) at 10 μg/ml diluted in sterile phosphate buffered saline (PBS; Fisher Scientific) and sterile carbonate bicarbonate (Sigma Aldrich) for 8 to 48 hours at 4°C. PBMCs were thawed and rested for 3-6 hours in RPMI media (Sigma) supplemented with 10% (v/v) Fetal Bovine Serum (Sigma), 1% (v/v) L-Glutamine (Sigma) and 1% (v/v) Penicillin/Streptomycin (Sigma) at 37°C, prior to stimulation with peptides. The capture antibody coated plates were washed four times with sterile PBS, then blocked with RPMI media supplemented with 10% (v/v) Fetal Bovine Serum and 1% (v/v) penicillin/streptomycin for two hours at 37°C. Overlapping peptide pools (18-mers with 10 amino acid overlap. Mimotopes) representing the spike (S), Membrane (M) or nucleocapsid (N) SARS-CoV-2 proteins were added to 250,000 PBMCs/well at a final concentration of 2 μg/ml for 16 to18 hours. For selected individuals, pools representing the S1 and S2 subunits of variant of concern were also included (B.1.35/beta and P.1/gamma). Pools consisting of CMV, EBV and influenza peptides at a final concentration of 2μg/ml (CEF; Proimmune) and phytohemagglutinin-L (Sigma) were used as positive controls. DMSO was used as the negative control at the equivalent concentration to the peptides. Wells were then washed with PBS with 0.05% (v/v) Tween20 (Sigma Aldrich) and incubated with the ELISpot Basic kit biotinylated detection antibody (clone 7-B6-1) diluted in PBS with 0.05% (v/v) Tween20 at 1 μg/ml, for 2 hours at room temperature. Wells were then washed with PBS with 0.05% (v/v) Tween20, and then incubated with the ELISpot Basic kit streptavidin-ALP, diluted in PBS at 1 μg/ml for 1.5 hours at RT. Wells were then washed with PBS and color development was carried out using the 1-step NBT/BCIP Substrate Solution. 50ul of filtered NBT/BCIP was added to each well for 15 minutes at RT. Color development was stopped by washing the wells with tap water. Air dry plates were scanned and analyzed with the AID Classic ELISpot reader (software version 8.0, Autoimmune Diagnostika GmbH, Germany). Antigen-specific responses were quantified by subtracting the mean spots of the control wells from the test wells and the results were expressed as spot-forming units (SFU)/10^6^ PBMCs.

#### Intracellular cytokine staining

T cell responses in selected IFNγ ELISpot positive samples were characterized further using intracellular cytokine staining (ICS) after stimulation with overlapping spike peptide pools. In brief, 1-1.5 × 10^6^ cells were plated in RPMI media (Merck) supplemented with 10% (v/v) Fetal Bovine Serum (ThermoFisher), 1% (v/v) L-Glutamine (Sigma) and 1% (v/v) Penicillin/Streptomycin (Sigma) and co-stimulatory antibodies; anti-CD28 (BD)and anti-CD49d (BD) in a 96 well U-bottom plate and peptide pools were added at 2 μg/ml final concentration for each peptide. DMSO was used as the negative control at the equivalent concentration to the peptides. As a positive control, cells were simultaneously stimulated with ionomycin (Sigma) at 500ng/ml and PMA (Sigma) at 50ng/ml final concentrations. Degranulation of T cells, a functional marker of cytotoxicity (Betts et al., 2003), was measured by the addition of an anti-CD107a specific antibody (BD) at 1 in 20 dilution during the culture. The cells were then incubated at 37°C, 5% CO_2_ for 1 hour before adding 10ug/ml Brefeldin A (Merck). Samples were incubated at 37°C, 5% CO_2_ for a further 5 hours before proceeding with staining for flow cytometry.

First, stimulated cells were stained with live/dead stain (ThermoFisher) 1:500 at RT in the dark for 20 minutes then washed in DPBS (ThermoFisher) followed by spinning the samples at 300 g for 5 minutes. Cells were then fixed in 2% formaldehyde (Merck) for 20 minutes, then frozen at −80°C in DPBS supplemented with 1% (v/v) bovine serum albumin (Merck), 10% (v/v) DMSO (Sigma). Cells were then thawed in batches, centrifuged at 400 g for 5 minutes to remove the freezing mix before permeabilization in 1x Perm/Wash buffer (BD) for 20-25 minutes at RT. Staining was performed in the dark at RT for 30 minutes in 1x Perm/Wash buffer with the antibodies listed in Key resources table, then the cells were washed and resuspended in DPBS. The samples were run on a FacsCanto II cytometer and the data were analyzed using FlowJo software version 10 (Treestar). Gating strategy exemplified in Figure S4.

### Quantification and statistical analysis

Continuous variables are displayed with median and interquartile range (IQR). Paired comparisons were performed using the Wilcoxon matched pairs signed rank test. Unpaired comparisons across two groups were performed using the Mann-Whitney test. Two-tailed P values are displayed. Statistical analyses were done using R version 3.5.1 and GraphPad Prism 9.0.1.

#### Statistical regression models

Multivariate regression models were created to estimate the associations between variables in the study cohort and antibody and T cell immune response. Variables included age, sex, ethnicity, previous infection, time point and vaccine dosing interval. Interactions and co-linearity between variables were explored and variables analyzed in separate models where necessary. Generalized linear models were created to estimate associations between the variables sex (discrete), age (continuous), Ethnicity (discrete), previous infection (discrete), and vaccine interval regimen (discrete) on spike ELISpot response (spike B SFU/10^6^; log transformed) or spike IgG response (SARS-Cov-2 S AU/ml; log transformed). Linear mixed-effect models were created to estimate associations between variables sex (discrete), age (continuous), sample time point (discrete), previous infection (discrete) and Ethnicity (discrete). on spike ELISpot response (spike B SFU/10^6^; log transformed) or spike IgG response (SARS-Cov-2 S AU/ml; log transformed) in data from the Long dosing interval. Interactions were found between previous infection and vaccine dosing interval in model 1 and 2 for Spike IgG responses, so separate models were run for naive and previously infected individuals. GLM and LMER models were performed in R /R studio. Summary tables were reported. To check assumptions were met, residuals versus fitted and Normal Q-Q diagnostic plots were created.

Model 1 < - glm (immune response ∼age + sex + previous infection + vaccine dosing interval + previous infection: vaccine dosing interval, data = data)
Model 2 < - glm (immune response ∼age + sex + previous infection + vaccine dosing interval + ethnicity + previous infection: vaccine dosing interval, data = data)
Model 3 < - lmer (immune response ∼age + sex + previous infection + sample time point + previous infection: time point, data = data)

## Data Availability

• IFN ELISpot data, MSD data, ACE2 inhibition data, neutralizing antibody data and intracellular cytokine assay data derived from human samples have been deposited at Mendeley Data and are publicly available as of the date of publication. The DOI is listed in the key resources table. • De-identified participant metadata have been deposited with the above Mendeley data and are publicly available as of the date of publication. • This paper does not report original code. • Any additional information required to reanalyse the data reported in this paper is available from the lead contact upon request.
