# Supplementary material for: Immunogenicity of standard and extended dosing intervals of BNT162b2 mRNA vaccine
Source: Cell. 2021 Nov 11;184(23):5699–5714.e11. doi: 10.1016/j.cell.2021.10.011 (PMC8519781; doi:10.1016/j.cell.2021.10.011)
Supplement: Document S1. Tables S1 and S2 [file mmc1.pdf]

Cell, Volume *184*

Supplemental information

Immunogenicity of standard and extended dosing intervals of BNT162b2 mRNA vaccine

Rebecca P. Payne, Stephanie Longet, James A. Austin, Donal T. Skelly, Wanwisa Dejnirattisai, Sandra Adele, Naomi Meardon, Sian Faustini, Saly Al-Taei, Shona C. Moore, Tom Tipton, Luisa M. Hering, Adrienn Angyal, Rebecca Brown, Alexander R. Nicols, Natalie Gillson, Susan L. Dobson, Ali Amini, Piyada Supasa, Andrew Cross, Alice Bridges-Webb, Laura Silva Reyes, Aline Linder, Gurjinder Sandhar, Jonathan A. Kilby, Jessica K. Tyerman, Thomas Altmann, Hailey Hornsby, Rachel Whitham, Eloise Phillips, Tom Malone, Alexander Hargreaves, Adrian Shields, Ayoub Saei, Sarah Foulkes, Lizzie Stafford, Sile Johnson, Daniel G. Wootton, Christopher P. Conlon, Katie Jeffery, Philippa C. Matthews, John Frater, Alexandra S. Deeks, Andrew J. Pollard, Anthony Brown, Sarah L. Rowland-Jones, Juthathip Mongkolsapaya, Eleanor Barnes, Susan Hopkins, Victoria Hall, Christina Dold, Christopher J.A. Duncan, Alex Richter, Miles Carroll, Gavin Screaton, Thushan I. de Silva, Lance Turtle, Paul Klenerman, Susanna Dunachie, and on behalf of the PITCH Consortium

Table S1. Overview of assays performed and number of samples tested. Related to Figure 2-5.

	Total participants (n=589)						
	Naïve (n=334)			Previously infected (n=255)			
	2-dose +4 weeks	Unpaired timecourse	Paired timecourse	2-dose +4 weeks	Unpaired timecourse	Paired timecourse	**Total**
SARS-CoV-2 spike IFN-gamma ELISpot	37 short<br>188 long	47 short<br>229 long	0 short<br>26 long	20 short<br>124 long	26 short<br>139 long	0 short<br>26 long	**83 short<br>368 long**
SARS-CoV-2 spike antibody (MSD[a])	41 short<br>151 long	51 short<br>183 long	0 short<br>29 long	19 short<br>169 long	29 short<br>199 long	0 short<br>29 long	**80 short<br>382 long**
Variant of Concern antibody (ELISpot)	0 short<br>40 long	0	0	0 short<br>42 long	0	0	**0 short<br>82 long**
Variant of Concern antibody (MSD[a])	23 short<br>94 long	0	0	14 short<br>118 long	0	0	**37 short<br>212 long**
Variant of Concern antibody (Neutralisation)	19 short<br>20 long	25 short<br>20 long	0	0	0	0	**25 short<br>20 long**
SARS-CoV-2 spike T cell intracellular cytokine staining	23 short<br>30 long	0	0	14 short<br>19 long	0	0	**37 short<br>49 long**
SARS-CoV-2 spike IgG ELISpot	12 short<br>10 long	0 short<br>10 long	0	0	0	0	**12 short<br>10 long**

[a] MSD = Mesoscale Discovery (MSD) binding assays

Table S2. Generalized linear models (GLM). Table shows three GLM models of T cell (naïve and previously infected individuals), antibody (naïve individuals) and antibody (previously infected individual) responses at 4 weeks after second dose. Variables include age, sex, previous infection, Ethnicity and vaccine dose interval. Variable references are Sex ; F (Female) versus M (Male), Previously infected; Yes versus No, Ethnicity; White versus Black/Asian/Mixed/Other, and Vaccine dose interval; Short versus Long. Related to Figure 3.

	**Spike T cell responses in naive and previously infected participants at dose-2 plus 4 weeks**			**Spike Antibody responses in naive participants at dose-2 plus 4 weeks**			**Spike Antibody responses in previously infected participants at dose-2 plus 4 weeks**		
Coeffcient	*Estimates*	*Conf. Int (95%)*	*P-Value*	*Estimates*	*Conf. Int (95%)*	*P-Value*	*Estimates*	*Conf. Int (95%)*	*P-Value*
Intercept	2.38	2.03 – 2.73	**<0.001**	5.39	5.14 – 5.65	**<0.001**	5.53	5.22 – 5.85	**<0.001**
Age	-0.01	-0.01 – 0.00	0.060	-0.01	-0.01 – -0.00	0.051	0.00	-0.00 – 0.00	0.926
Sex (M)	-0.04	-0.20 – 0.13	0.683	-0.07	-0.19 – 0.05	0.232	0.05	-0.08 – 0.17	0.476
Previously infected (Yes)	0.36	0.19 – 0.52	**<0.001**						
Ethnicity (Black)	0.18	-0.55 – 0.91	0.623	0.05	-0.63 – 0.72	0.893	0.13	-0.19 – 0.46	0.416
Ethnicity (Asian)	-0.16	-0.42 – 0.09	0.203	0.05	-0.16 – 0.27	0.645	0.10	-0.08 – 0.28	0.258
Ethnicity (Mixed)	-0.16	-0.68 – 0.37	0.560	0.42	-0.25 – 1.10	0.223	-0.63	-1.23 – -0.03	**0.043**
Ethnicity (Other)	-0.05	-0.57 – 0.48	0.867	-0.18	-0.57 – 0.21	0.374	0.16	-0.16 – 0.49	0.326
Vaccine dose interval (Long)	-0.20	-0.42 – 0.01	0.060	0.19	0.05 – 0.32	**0.007**	-0.01	-0.24 – 0.22	0.908
Observations	277			143			139		
R^2	0.085			0.131			0.058		
